# Modulation of Aging Diseases via RAGE Targets: A Dietary Intervention Review

**DOI:** 10.1002/advs.202510242

**Published:** 2025-08-21

**Authors:** Qian Wu, Jia Yan, Yuyan Zhang, Xiaozhi Ming, Siqi Chen, Zhichao Zou, Nianjie Feng, Jianbo Xiao

**Affiliations:** ^1^ Cooperative Innovation Center of Industrial Fermentation (Ministry of Education & Hubei Province) Key Laboratory of Fermentation Engineering (Ministry of Education) National “111” Center for Cellular Regulation and Molecular Pharmaceutics Hubei Key Laboratory of Industrial Microbiology Hubei University of Technology Wuhan Hubei 430068 China; ^2^ Faculty of Sciences Department of Analytical and Food Chemistry Universidade de Vigo Nutrition and Bromatology Group Ourense 32448 Spain

**Keywords:** aging diseases, dietary, interaction, modulation, RAGE

## Abstract

With the acceleration of global population aging, effective strategies for the prevention and management of aging‐related diseases have become increasingly urgent. The receptor for advanced glycation end products (RAGE), a pattern recognition receptor of the immunoglobulin superfamily, plays a central regulatory role in the pathogenesis of chronic conditions such as diabetes and Alzheimer's disease. By binding to a wide range of ligands (e.g., advanced glycation end products, amyloid beta), RAGE activates key inflammatory and stress‐related signaling pathways, including NF‐κB and MAPK, positioning it as a critical therapeutic target. This review systematically examines RAGE‐ligand interactions and their downstream signaling cascades, and proposes targeted intervention strategies. Special emphasis is placed on the regulatory potential of dietary bioactive compounds, such as polyphenols, polysaccharides, and terpenoids, highlighting the distinct advantages of functional foods in anti‐aging applications. In line with the World Health Organization's concept of “preventive aging,” dietary‐based approaches offer a long‐term, safe, and integrative means of providing both nutritional support and disease prevention. This review provides a theoretical foundation for the development of RAGE‐targeted dietary interventions and supports a paradigm shift from medical treatment to nutritional prevention in anti‐aging strategies.

## Graphical abstract

This paper analyzes RAGE ligand‐binding mechanisms and signaling, proposing intervention strategies targeting these interactions. It emphasizes dietary polyphenols, polysaccharides, and terpenoids delivered via functional foods for anti‐aging. These compounds offer long‐term, safe intervention through dietary supplementation, providing dual nutritional support and disease prevention benefits.

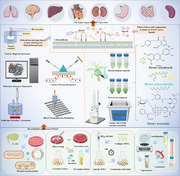

## 1 Introduction

**Figure 1:**
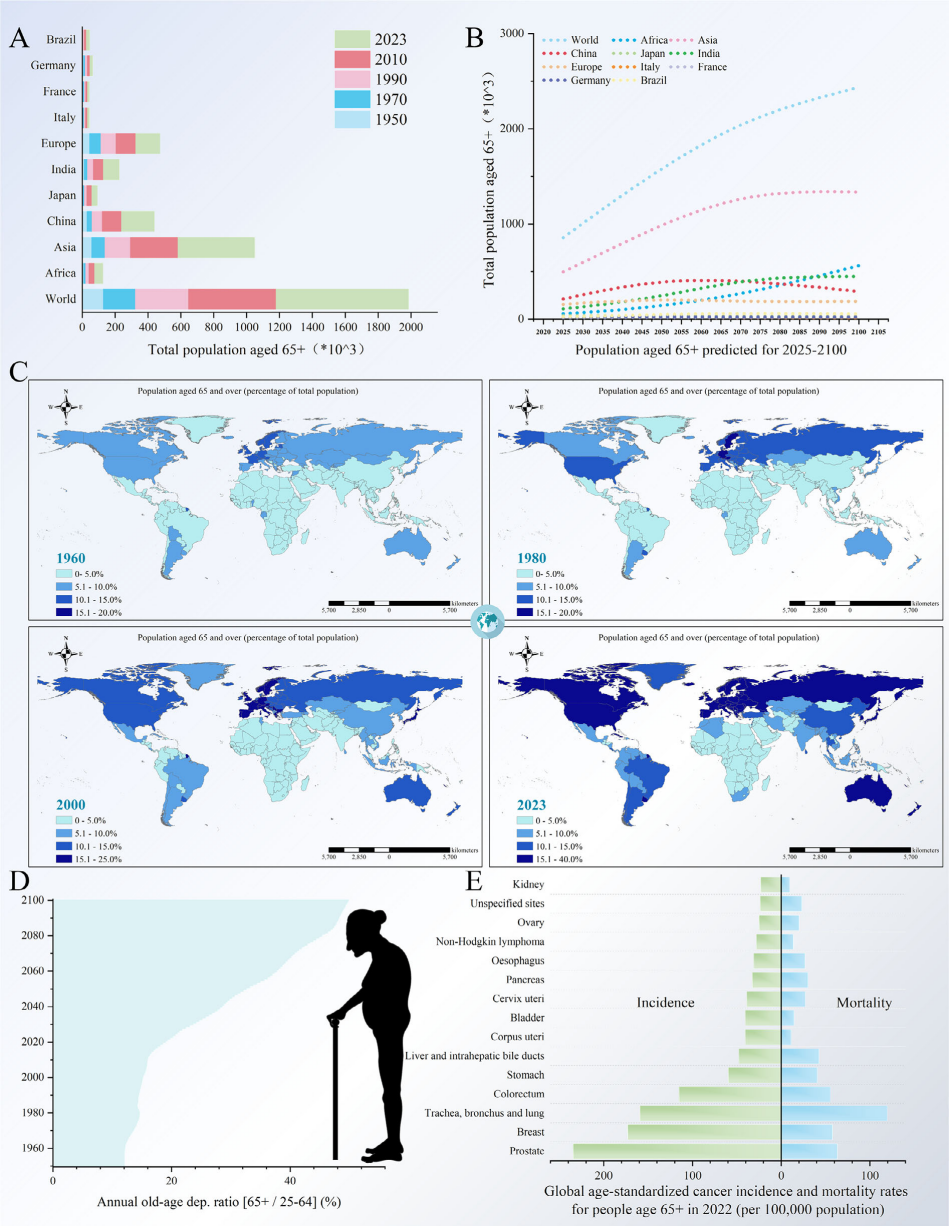
World population ageing trends and challenges. A) Growth of the world population aged 65 years and over from 1950 to 2023. B) Projected growth of the world population aged 65 years and over from 2025 to 2100. C) Distribution of the world population aged 65+ from 1960 to 2023. D) Estimated annual dependency ratios for age 65+ from 1950 to 2100. E) Global age‐standardized cancer incidence and mortality rates for people aged 65 years and older in 2022.

Global population aging has become a pressing challenge, with developed nations such as Japan (28% aged 65 and older in 2023) and Italy (24%) experiencing significant demographic shifts, while developing countries like China anticipate their elderly population to triple by 2050. According to United Nations projections, the global population aged 60 and older is expected to double to 2.1 billion (21.3% of the total population) by 2050, driven by increasing life expectancy, which has risen from a global average of 46 years in 1950 to 73 years in 2020. The immediate consequences of population aging include a shrinking labor force, increased pressure on social security systems, and a surge in demand for healthcare resources. Even more concerning, aging not only exacerbates the economic burden but also leads to a higher incidence of numerous chronic diseases associated with aging, such as cardiovascular diseases, diabetes, and Alzheimer's disease (AD).^[^ ^1^ ^]^ The prevalence of these conditions not only diminishes the quality of life for the elderly but also presents significant challenges to global healthcare systems (**Figure** **1**).

Receptor for Advanced Glycation End Products (RAGE) and its diverse ligands play a pivotal role in the mechanisms underlying aging‐related diseases. RAGE is a transmembrane protein with a molecular weight of 50–55 kDa, classified within the immunoglobulin (Ig) superfamily. The gene responsible for encoding human RAGE is situated on chromosome 6, within the class III major histocompatibility complex, and comprises 11 exons interspersed with 10 shorter introns.^[^ ^2^ ^]^ RAGE, as a central signaling molecule in the innate immune system, plays a crucial role in regulating inflammation. Its expression is spatiotemporally specific: it is widely distributed across various tissues during embryonic development, but in adulthood, it exhibits tissue‐selective, low expression, with notably high levels in lung tissues, particularly in type I alveolar epithelial cells. This distinctive expression pattern suggests that RAGE may have an important physiological role in maintaining alveolar structural stability, as well as in the maturation and differentiation of epithelial cells. Importantly, aberrant expression of RAGE in the lungs has been closely linked to the development of major respiratory diseases, including lung cancer, pulmonary fibrosis, and chronic obstructive pulmonary disease.^[^ ^3^ ^]^

Capable of interacting with diverse molecules lacking sequence homology, RAGE recognizes ligands' 3D conformations rather than specific amino acid sequences. This enables RAGE to function as a pattern recognition receptor (PRR). Its broad‐spectrum binding property has positioned RAGE as a central player in key physiopathological processes, including cell signaling, inflammatory responses, and oxidative stress.^[^ ^4^ ^]^ Originally identified as a receptor for advanced glycation end products (AGEs), RAGE has since been implicated in a wide range of conditions, including vascular lesions, diabetes, neurodegenerative diseases, and various cancers. Its ligand profile includes several molecules such as high mobility group box 1 (HMGB1), S100, amyloid beta (Aβ), and amyloid polypeptide (IAPP), each associated with specific disease phenotypes.^[^ ^5^ ^]^ For example, AGEs‐RAGE interactions contribute to vascular endothelial dysfunction and atherosclerosis, while exacerbating insulin resistance in diabetes mellitus.^[^ ^6^, ^7^ ^]^ On the other hand, Aβ‐RAGE binding promotes neuroinflammatory processes in AD.^[^ ^8^ ^]^ Ligand binding triggers receptor activation, initiating cascade signaling that leads to pathological changes such as chronic inflammation and apoptosis, mechanisms that are especially prominent in aging‐related diseases.^[^ ^9^ ^]^ Thus, analyzing the RAGE signaling network not only helps to uncover the molecular mechanisms underlying aging but also provides a theoretical foundation for the development of early diagnostic markers and targeted therapeutic strategies. As such, RAGE represents an important potential target for interventions in age‐related diseases. Among the promising and relatively novel non‐pharmacological strategies being explored to modulate this target are naturally occurring bioactive dietary components.

However, structural studies of RAGE, a key target in aging regulation, face several challenges. These include the inherent tendency of membrane proteins to oligomerize, the highly dynamic nature of their structural domains, and their dependence on detergents and lipids to maintain solubility, all of which complicate their biophysical analysis.

Critically, the predisposition of RAGE to drive disease is intimately linked to its ligand‐binding characteristics. The affinity of RAGE for its ligands, which dictates signaling outcomes, is determined by both the association (Kon) and dissociation (Koff) rates, governed by binding stability and occupancy of the receptor's binding cavity.^[^ ^10^ ^]^ Receptor‐ligand interactions like these underpin fundamental processes in cellular communication, tissue development, and diverse physiopathologies, including cancer metastasis. Consequently, analyzing and modulating these interactions is paramount for understanding disease mechanisms and developing effective therapeutic strategies.^[^ ^11^ ^]^ In recent years, the dynamic analysis of receptor‐ligand complex lifetimes, quantified by Kon and residence time (1 Koff^−1^), has become a major research focus. A promising strategy to modulate these kinetics is through heterotopic (allosteric) ligands, which bind to non‐orthosteric sites on the receptor, inducing conformational changes that can positively or negatively influence the binding kinetics of the endogenous ligand.^[^ ^12^, ^13^, ^14^ ^]^ While this allosteric modulation strategy has been extensively explored for targets like G protein‐coupled receptors (GPCRs), it remains significantly underexplored in the context of RAGE‐ligand interactions, representing a key knowledge gap and opportunity.

This gap highlights the significant potential and novelty of exploring naturally occurring bioactive dietary components as potential allosteric modulators of RAGE.^[^ ^15^, ^16^, ^17^, ^18^ ^]^ Targeting RAGE signaling through dietary bioactives offers a promising and distinct non‐pharmacological strategy. Functional foods present compelling advantages over conventional drugs in anti‐aging interventions, including higher biosafety, lower risk of toxicity and side effects, and the potential for long‐term, sustained modulation through routine consumption.^[^ ^19^ ^]^ The exploration of dietary bioactives for RAGE modulation represents an active and evolving area of research with significant potential for preventing age‐related chronic diseases.^[^ ^20^ ^]^

In this review, we systematically propose a strategy for analyzing RAGE interactions, integrating advances in ligand binding kinetics to elucidate molecular mechanisms underlying aging‐related diseases. We review the role of dietary polyphenols, polysaccharides, and terpenoids in modulating the RAGE signaling pathway through multi‐targeted interventions. These natural bioactive ingredients not only inhibit RAGE‐ligand interactions directly, but also achieve sustained modulation through various mechanisms, including the inhibition of ligand production, thereby offering a comprehensive approach to disease prevention and management. This review provides a theoretical foundation for the development of novel functional foods that combine both nutritional benefits and targeted intervention properties by elucidating the RAGE molecular interaction network. It is important to highlight that bioactive substances found in functional foods often exert synergistic effects due to their multicomponent nature, a mode of action that aligns more closely with the complex pathological characteristics of aging than single‐target drugs. The strategy of inhibiting RAGE through dietary components opens a promising new avenue for the prevention and treatment of aging‐related diseases. Future research should prioritize analyzing the structural basis of interactions between natural compounds and RAGE, while also establishing a quality standard system for functional food active ingredients. This will help drive the transformation of anti‐aging research toward precision nutritional intervention, enabling more effective and tailored approaches to combating age‐related diseases.

## 2 Structure and Recombinant Expression of RAGE

### 2.1 Structure of RAGE

The mature RAGE protein consists of 404 amino acids and is structurally organized into three main segments: the intracellular segment, the extracellular segment, and the transmembrane segment. These segments include five structural domains: three extracellular Ig‐like domains, a variable, positively charged V domain, a positively charged C1 domain, and a negatively charged C2 domain, a transmembrane domain, and a 43‐amino acid C‐terminal cytoplasmic tail (ctRAGE).^[^ ^21^ ^]^ The extracellular fragment is the primary binding site for ligands, while ctRAGE binds to adaptor proteins, such as diaphanous‐related formin 1 (DIAPH1) and the toll‐interleukin 1 receptor domain‐containing adaptor protein, to activate downstream signaling pathways.^[^ ^22^ ^]^ Notably, the intracellular domains lack sequences homologous to any known signaling regions. RAGE also contains two N‐glycation sites that are critical for ligand binding, one located within the V domain and another positioned nearby. These glycation sites contribute to the formation of RAGE oligomers on the cell surface, which are important for receptor function.^[^ ^23^ ^]^

Recent cryo‐electron microscopy (cryo‐EM) analysis has provided further insights into the ligand‐binding mechanism of RAGE. Kim et al. resolved the structure of the RAGE–HMGB1 complex at 5.19 Å resolution, revealing that the V domain of RAGE, specifically residues P66, G70, P71, S74, and R77, forms a positively charged binding pocket that engages HMGB1 via electrostatic interactions with its acidic residues (E145, K146, E153, E156). This structural evidence substantiates the hypothesis that the V domain functions as an “electrostatic trap” for anionic ligands such as HMGB1, aligning with prior biochemical characterizations. Supporting this, site‐directed mutagenesis demonstrated that alanine substitutions at key basic residues (R70 and R77) significantly impaired HMGB1 binding, further validating the critical role of these interactions in ligand recognition.^[^ ^24^ ^]^

**Figure 2:**
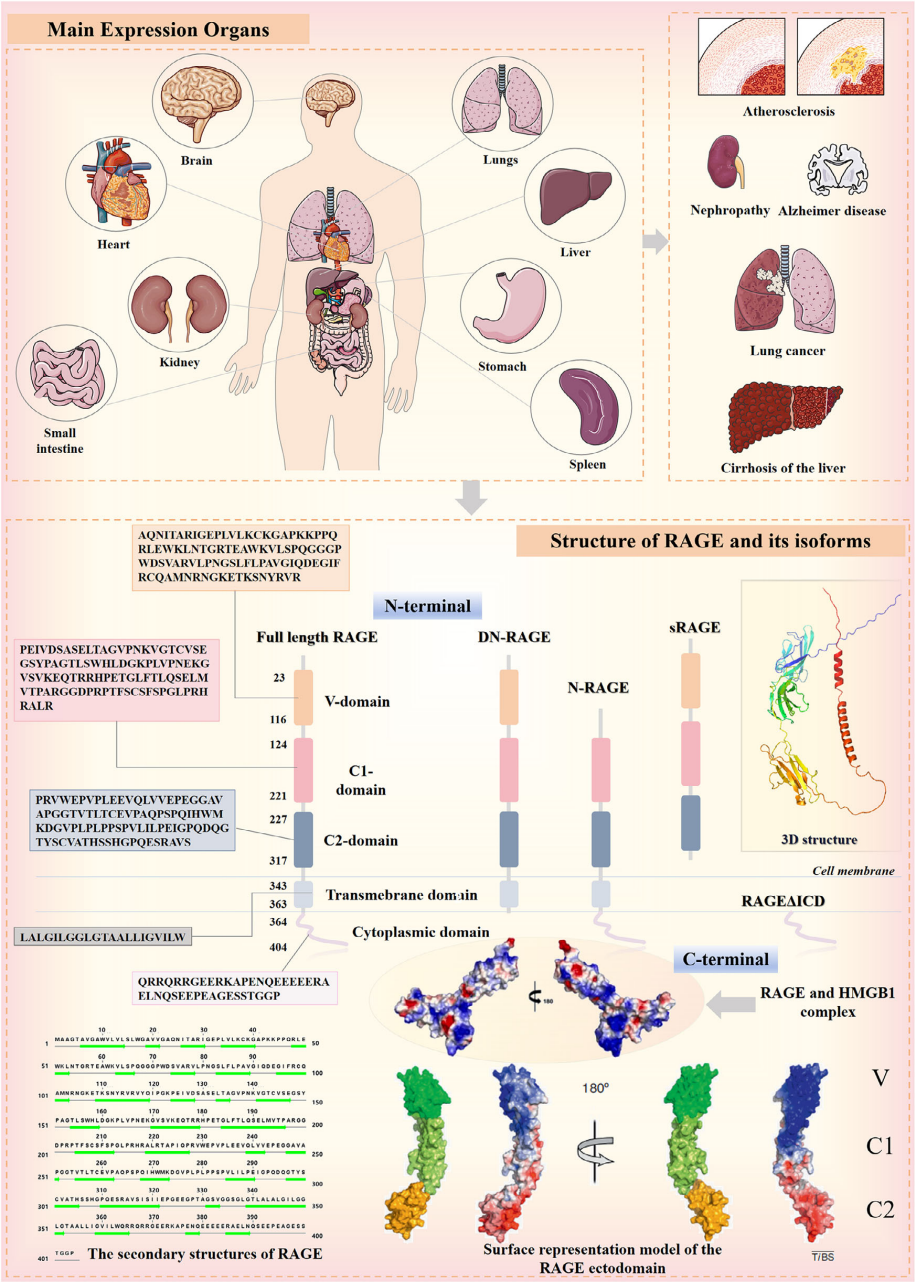
Organ of RAGE expression and its structure and isoforms. The main organs where RAGE is expressed are the heart, lungs, and brain, and it causes diseases such as atherosclerosis and Alzheimer's disease, etc. The isoforms of RAGE are DN‐RAGE, N‐RAGE, sRAGE, and RAGEΔICD. The structure of RAGE is shown with its secondary structure and Surface representation model of its extracellular structural domains (Reproduced with permission.^[^ ^55^ ^]^ Copyright 2011 Elsevier Ltd.), along with isosurfaces reconstructed by cryo‐EM of the RAGE and HMGB1 complexes (Reproduced with permission.^[^ ^24^ ^]^ Copyright 2025 Elsevier Masson SAS.).

Depending on the splicing position, several RAGE isoforms can be generated (**Figure** **2**). For instance, N‐truncated RAGE (N‐RAGE) lacks the V domain, thereby disrupting ligand interactions within this region. Conversely, dominant‐negative RAGE (DN‐RAGE) lacks a cytoplasmic region, which prevents activation of key downstream signaling pathways. Soluble RAGE (sRAGE) consists solely of the extracellular domain, produced through the selective cleavage of full‐length RAGE by GPCRs or matrix metalloproteinases (MMPs). Like DN‐RAGE, sRAGE lacks cytoplasmic activity and does not possess the biological capacity to interact directly with RAGE ligands. Instead, sRAGE functions to clear RAGE ligands from the extracellular matrix (ECM) and acts as a decoy receptor, effectively sequestering these ligands and blocking their interaction with membrane‐bound RAGE.^[^ ^25^ ^]^ The decoy function of sRAGE is structurally substantiated: cryo‐EM analysis demonstrates that the isolated V domain of sRAGE preserves the key HMGB1‐binding residues (P66–R77).^[^ ^24^ ^]^ This structural conservation explains its capacity to competitively inhibit HMGB1–RAGE signaling by sequestering ligands through the same binding interface utilized by membrane‐bound RAGE. Another isoform, RAGEΔICD, characterized by a truncated intracellular domain, is predominantly found in mouse and human tissues, including the heart, kidneys, brain, and lungs. This variant exhibits dominant‐negative effects, modulating RAGE cell signaling pathways and attenuating RAGE‐mediated biological effects.^[^ ^26^ ^]^

RAGE exists as a weakly constitutive oligomer, with each extracellular structural domain containing two cysteine residues that participate in intramolecular disulfide bond formation. The cysteines in the C2 region also facilitate intermolecular bridges that stabilize the RAGE dimer. Structural investigations of the extracellular portion of RAGE, including the V, C1, and C2 domains, have demonstrated that the V and C1 domains create an elongated structural unit linked to the C2 domain via a flexible linker. Oligomerization, a widespread phenomenon observed in at least two‐thirds of cellular proteins, is frequently vital for efficient interactions between membrane proteins and their ligands, playing a key role in signal transduction processes. However, the exact mechanism underlying RAGE oligomerization remains a topic of controversy. One hypothesis posits that RAGE undergoes ligand‐dependent oligomerization, while another suggests that RAGE is preassembled into oligomeric complexes prior to ligand binding through interactions with acetylheparan sulfate (HS), a negatively charged polysaccharide commonly found on cell surfaces. This latter hypothesis is reinforced by detailed structural analyses, including crystallographic and solution studies, which demonstrate that HS oligosaccharides can promote the formation of stable RAGE hexamers even in the absence of ligands.^[^ ^27^, ^28^ ^]^ Cryo‐EM structural data further support the hypothesis that ligand binding promotes RAGE oligomerization. Reconstructions of the RAGE–HMGB1 complex revealed notable conformational flexibility, capturing three distinct oligomeric states.^[^ ^24^ ^]^ This structural plasticity may underlie RAGE's ability to accommodate a wide spectrum of ligands such as S100 proteins and AGEs, which exist within the same V‐structural domain binding pocket. However, the precise nature of ligand‐specific binding modes and their contributions to downstream signaling remain to be fully elucidated.

### 2.2 Recombinant Expression of RAGE

**Figure 3:**
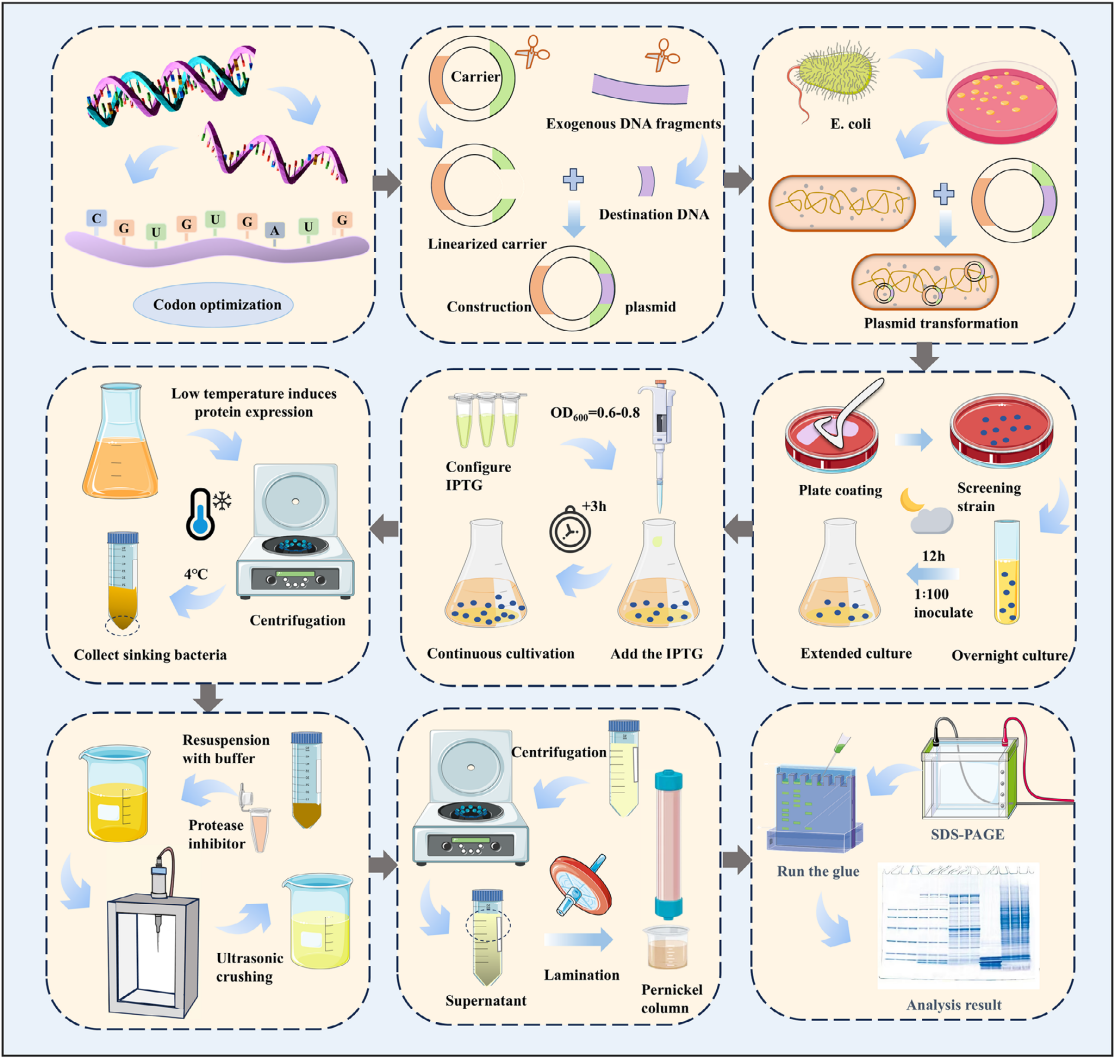
Flowchart of RAGE expression in E. coli expression system. The main process of RAGE expression and purification in E. coli is codon optimization, construction of plasmid, transformation into E. coli, screening, expanded culture, induction of expression, purification, and validation.

RAGE, as a protein in the human body, can be produced using a protein expression system, a molecular biology technique designed for recombinant protein production. The process of obtaining recombinant proteins is well established and typically involves several key steps: constructing a plasmid, inserting the target protein gene into the desired plasmid vector, transforming the plasmid into a suitable host organism, inducing protein expression, and finally purifying the protein using various separation methods and characterizing it. However, in practical experiments, unsuccessful expression is not uncommon, often arising from challenges such as low expression levels, difficulties in achieving soluble protein, or inherent issues associated with membrane proteins. As RAGE is classified among the difficult‐to‐express proteins, it poses particular challenges. While numerous studies have proposed solutions to expression problems for various protein classes, the specific obstacles associated with the expression and purification of RAGE have not yet been comprehensively summarized. Therefore, this article focuses on several cases of RAGE expression in different systems, aiming to outline the process (**Figure** **3**) and highlight important precautions.

#### 2.2.1 Selection of Host System

The organisms used in protein expression systems can include bacteria, yeast, plant cells, and animal cells. Selecting an appropriate expression host is crucial for achieving efficient protein expression. Prokaryotic expression systems, with *Escherichia coli* (E. coli) being the most widely used, are both cost‐effective and commonly employed. This system offers several advantages, including a clear genetic background, rapid growth, the ability to achieve high cell density cultures, straightforward transformation of exogenous DNA, low operational costs, high expression yields, and relatively simple isolation and purification of the expressed product.^[^ ^29^, ^30^ ^]^ However, there are notable disadvantages to using E. coli. The primary limitation is the lack of post‐translational processing mechanisms, including glycation, disulfide bond formation, and proper protein folding, which reduces the likelihood of obtaining biologically active proteins. In contrast, yeast, as a eukaryotic organism, has a glycation mechanism that is more similar to that of higher eukaryotes, making it more likely for the expressed proteins to retain biological activity. The yeast protein expression system also boasts several advantages, including high expression levels, inducibility, ease of purification for secreted proteins, and the ability to achieve high‐density fermentation.

However, some protein products can be prone to degradation, and the expression levels may not be easily controllable. Therefore, it is essential to select an appropriate host for RAGE expression based on its specific properties and experimental requirements. Existing studies have successfully expressed RAGE using Pichia pastoris and BL21‐derived E. coli.^[^ ^31^ ^]^ Currently, E. coli‐based expression systems account for a significant proportion of the yield of difficult‐to‐express proteins. They have become the preferred heterologous expression system due to their relative simplicity and cost‐effectiveness. Additionally, E. coli is characterized by a wide range of commercial vectors, well‐defined genetic information, various mutant strains, and numerous gene‐editing tools.^[^ ^32^ ^]^ In summary, this paper focuses on detailing the circumstances under which RAGE has been expressed in E. coli.

The first step in the expression of RAGE involves the selection of appropriate E. coli strains. Since RAGE is a eukaryotic protein and E. coli is a prokaryotic expression system, some tRNAs present in eukaryotes may be absent during RAGE expression. Consequently, ≈30% of RAGE proteins in E. coli may misfold due to the absence of necessary cellular mechanisms, leading to protein aggregation in the cytoplasm.^[^ ^33^ ^]^ To address these challenges, researchers have developed various E. coli strains, including Rosetta‐gami (DE3), Origami‐B (DE3), C41 (DE3), and C43 (DE3), which are better suited for the soluble expression of difficult‐to‐express proteins. These strains are engineered to improve solubility and facilitate the proper formation of disulfide bonds.^[^ ^34^ ^]^ When expressing RAGE, strains like Rosetta or BL21‐CodonPlus, which contain rare codons, are often chosen. These specialized strains can improve the soluble expression levels of RAGE in prokaryotic systems, thereby facilitating more effective protein production (**Table** **1**).

**Table 1:** Recombinant expression and purification of RAGE in E. coli.

Structural domain	E. coli host	Plasmid Type	Purification Tags	Tag Position	Resistance	Purification methods	Refs.
**C2**	E. coli strain BL21(DE3) codon plus	pET‐28a	6His	N	Kanamycin	Cobalt column and Superdex 75 column	[38]
**V/V‐C1**	E. coli BL21(DE3) Origami B		6His	N		Ni‐Sepharose Fast Flow column and Superdex 75 column	[207]
**sRAGE, RAGE, V**	BL21 E. coli	pGEX‐4T‐1	GST		Ampicillin	MagneGST beads	[208]
**V, V‐C1, and V‐C1‐C2**	Shuffle T7 Express E. coli cells	pETM11	6His	N	Kanamycin	Ni‐column and Superdex 75 column	[209]
**V‐C1‐C2**	Origami‐B (DE3)	pET21b				HiTrap SP cation exchange column and Superdex 200 column	[27]
**V‐C1**	E. coli strain Origami B(DE3)	pET15b	6his	N	Ampicillin	Ni‐NTA Column and SE‐75 column	[85]
**V‐C1‐C2, V‐C1**	Shuffle T7 Express competent cells	pETM11	6His	N	Kanamycin	Ni‐NTA column	[210]
**full‐length RAGE**	E. coli strain BL21(DE3)	pET24	6His	C	Kanamycin	Ni Sepharose 6 Fast Flow slurry and HiTrap SP cation exchange column	[211]
**V**	E. coli BL21 (DE3) cells.	pET‐28a	6His	N		Ni‐NTA column and Superdex 75 gel filtration column	[212]
**hsRAGE**	E. coli strain Origami (DE3)	pETavi	6His	N and C		Ni–NTA column,	[213]
**V‐C1**	BL21 (DE3) Codon Plus RIPL cells	pET30a	GB1 tag	N	Kanamycin	HisPrep IMAC column, IMAC column, phenyl‐Sepharose hydrophobic interaction chromatography and size exclusion chromatography	[214]
**sRAGE, V‐C1, C1‐C2, V, C1, C2**	E. coli strain OrigamiB(DE3)	pET15b	6His tag	N		His‐Select resin	[215]
**V‐C1‐C2**	E. coli Shuffle T7 Express cells	pETM11	6His tag	N		Two‐step Ni‐column affinity chromatography and cation‐exchange chromatography	[216]

#### 2.2.2 Selection of Plasmid

The most commonly used expression plasmids incorporate various elements, including replicons, promoters, selection tags, multiple cloning sites, and fusion proteins or cleavage sites for these fusion proteins. As research progresses, an increasing number of expression vectors have become available, making it essential to select the appropriate plasmid based on specific experimental needs.^[^ ^35^ ^]^ For RAGE expression, the pET series vectors are among the most frequently employed plasmids. The PET vector system is a powerful and widely utilized platform for expressing recombinant proteins in E. coli.^[^ ^36^ ^]^ The presence of the phage T7 strong promoter in these vectors enables effective regulation of both transcription and translation of the target gene, facilitating high levels of protein expression.^[^ ^37^ ^]^ Vectors that have successfully been used to express RAGE include PET‐28a, PET‐15b, and others.^[^ ^38^ ^]^ Codon optimization is also a critical component of successful heterologous expression. This technology focuses on employing the codon adaptation index (CAI) to replace low‐abundance codons, alleviating translation elongation bottlenecks. A higher CAI value signifies fewer rare codons.^[^ ^39^ ^]^ Due to species‐specific codon preferences, particularly the scarcity of rare codons in E. coli, optimizing the codons for heterologous production can significantly enhance the stability and translation efficiency of the resulting mRNA.^[^ ^40^ ^]^ Consequently, researchers frequently focus on eliminating rare codons and optimizing GC ratios while considering ribosome binding site sequences to prevent the formation of undesirable hairpin structures.^[^ ^41^ ^]^ In addition to CAI, the mRNA adaptation index and tRNA usage frequency should be assessed using online tools, such as the Codon Usage Table in GenBank. Moreover, selecting an appropriate host bacterium remains another effective strategy for the production of heterologous proteins, complementing the codon optimization efforts discussed above.^[^ ^42^ ^]^

#### 2.2.3 Selection of Tags

A protein tag is a peptide or protein that is expressed in fusion with a target protein using DNA recombination technology, facilitating the expression, detection, tracking, and purification of the target protein. As technology has advanced, researchers have developed various protein tags with different functions. The choice of tag often depends on the specific experimental objectives. Commonly used tags include 6×His, maltose‐binding protein (MBP), thioredoxin, Flag, glutathione S‐transferase (GST), c‐Myc, eGFP/eCFP/eYFP/mCherry, HA, and SUMO.^[^ ^43^, ^44^, ^45^ ^]^ The primary role of the chaperone system is to recognize folded intermediates or misfolded proteins, assisting in the proper folding of target proteins and helping them to separate from the cytoplasmic environment. In the context of RAGE expression, most successful cases have utilized the 6×His tag. RAGE can sometimes be unstable when expressed in bacteria; therefore, the use of fusion proteins can enhance its stability and solubility, allowing for the expression of active and soluble target proteins. Fusion partners provide additional properties, including immunodetection, quantification, and the ability to study protein‐protein interactions in heterogeneous proteins.^[^ ^46^ ^]^ When screening for soluble tags, key characteristics to consider include the tag's properties, isoelectric point, molecular size, and the site of fusion.^[^ ^42^ ^]^ Several mechanisms for solubilization have been proposed:^[^ ^43^ ^]^ I. Formation of Micelle‐like Structures: These structures isolate and protect misfolded proteins from solvents or adverse conditions, such as high temperatures.^[^ ^47^ ^]^ II. Facilitating Target Protein Folding: Fusion partners can drive target proteins into a partner‐mediated folding pathway. For example, MBP in E. coli and NusA can interact with GroEL to assist in proper folding. III. Intrinsic Chaperonin‐like Activity: Some fusion partners possess intrinsic properties that assist in protein folding and inhibit self‐aggregation. IV. Electrostatic Repulsion: Highly acidic fusion partners can help prevent protein aggregation through electrostatic repulsion.^[^ ^48^ ^]^ These mechanisms enhance the stability and solubility of target proteins, ultimately aiding in their expression and purification.

#### 2.2.4 Selection of Purification Methods

There are three commonly used protein purification methods: affinity chromatography, ion exchange chromatography, and gel filtration chromatography, also known as molecular sieve chromatography.

Affinity chromatography is a separation method that exploits the specific binding interactions between target proteins and certain ligands. In this method, the target protein is attached to a stationary phase that contains a ligand to which it has a strong affinity. As a result, proteins that do not bind to the ligand are washed away, allowing the target protein to be purified in a single step with high specificity and purity. This technique is particularly effective when dealing with complex protein mixtures.^[^ ^49^ ^]^

Ion exchange chromatography separates proteins based on their charge. This method involves an ion exchange resin that can be either a cation exchange agent, such as carboxymethylcellulose, or an anion exchange agent, such as diethylaminoethylcellulose. When a protein solution is introduced into the column, proteins with an opposite charge to the ion exchanger will bind to the resin. The bound proteins can then be eluted by adjusting the pH or ionic strength of the elution buffer, allowing for their separation from unbound proteins.^[^ ^50^ ^]^

Gel filtration chromatography separates proteins based on their molecular size or shape. This method uses a porous gel matrix, which allows smaller molecules to enter the pores while excluding larger molecules. Consequently, larger proteins elute first, while smaller proteins are retained longer in the column. This technique is especially effective for separating proteins with a molecular weight difference of more than three times and can also be used for buffer exchange or desalting.^[^ ^51^ ^]^

These methods are commonly used in combination to achieve high‐purity protein preparations, making them essential tools in biochemistry and molecular biology. RAGE is frequently purified using affinity chromatography, with the commonly utilized label being 6×His. This tag specifically binds to nickel (Ni^2+^) columns, allowing for the efficient purification of RAGE. The elution of RAGE from the column can be achieved by gradually increasing the concentration of imidazole, which competes with the His‐tag for binding to the nickel, thereby releasing the target protein. In addition to affinity chromatography, ion exchange chromatography and gel filtration chromatography are also typically employed for further purification and fine separation, especially in structure‐related experiments. Together, these methods ensure that RAGE is sufficiently purified for subsequent structural determination and functional studies, enhancing the reliability of experimental outcomes.

#### 2.2.5 Other Influencing Factors

The expression of RAGE in E. coli is influenced by several critical factors, including culture medium composition, inducer concentration, cell density at induction, induction time, and induction temperature. Careful optimization of these parameters is essential for maximizing protein yield and solubility. Culture Medium: The choice of growth medium plays a significant role in protein expression. RAGE is commonly expressed in LB medium or M9 medium.^[^ ^29^ ^]^ The growth medium can be either complex, providing rich nutrients, or defined, allowing for precise control over the environment. Selecting an appropriate carbon source and any necessary supplements can further enhance growth and expression.^[^ ^52^ ^]^ Inducer Concentration: The most commonly used inducer for protein expression in E. coli is isopropyl β‐D‐1‐thiogalactopyranoside (IPTG). The concentration of IPTG needs to be optimized based on the specific strain and plasmid being used. Higher IPTG concentrations can sometimes lead to increased expression, but may also result in higher levels of aggregation or inclusion bodies.^[^ ^53^ ^]^ Cell Density at Induction: The cell density at which IPTG is added significantly affects expression levels. Generally, the optimal cell density for induction occurs in the log phase of growth, typically when the optical density at 600 nm (A_600_) reaches 0.6‐0.8. This range ensures that the cells are actively dividing and capable of high levels of protein synthesis.^[^ ^54^ ^]^ Induction Time: The duration of induction can also impact the yield and quality of the expressed protein. Typically, induction lasts several hours; however, this may vary depending on the specific requirements of RAGE and the expression system used. Induction Temperature: The temperature at which induction occurs influences the rate of cell growth and the folding of the recombinant protein. Generally, induction temperatures range between 16 and 30 °C, with lower temperatures often favored for enhancing protein solubility and reducing aggregation. The ideal temperature can depend on the plasmid used, as well as the properties of the expressed protein. To achieve optimal expression of RAGE, a systematic experimental approach should be employed to analyze these variables. By fine‐tuning each of these factors, researchers can maximize RAGE yield while minimizing issues related to protein aggregation and instability.^[^ ^53^ ^]^

## 3 Interaction of RAGE with Ligands and Its Binding Kinetics

**Figure 4:**
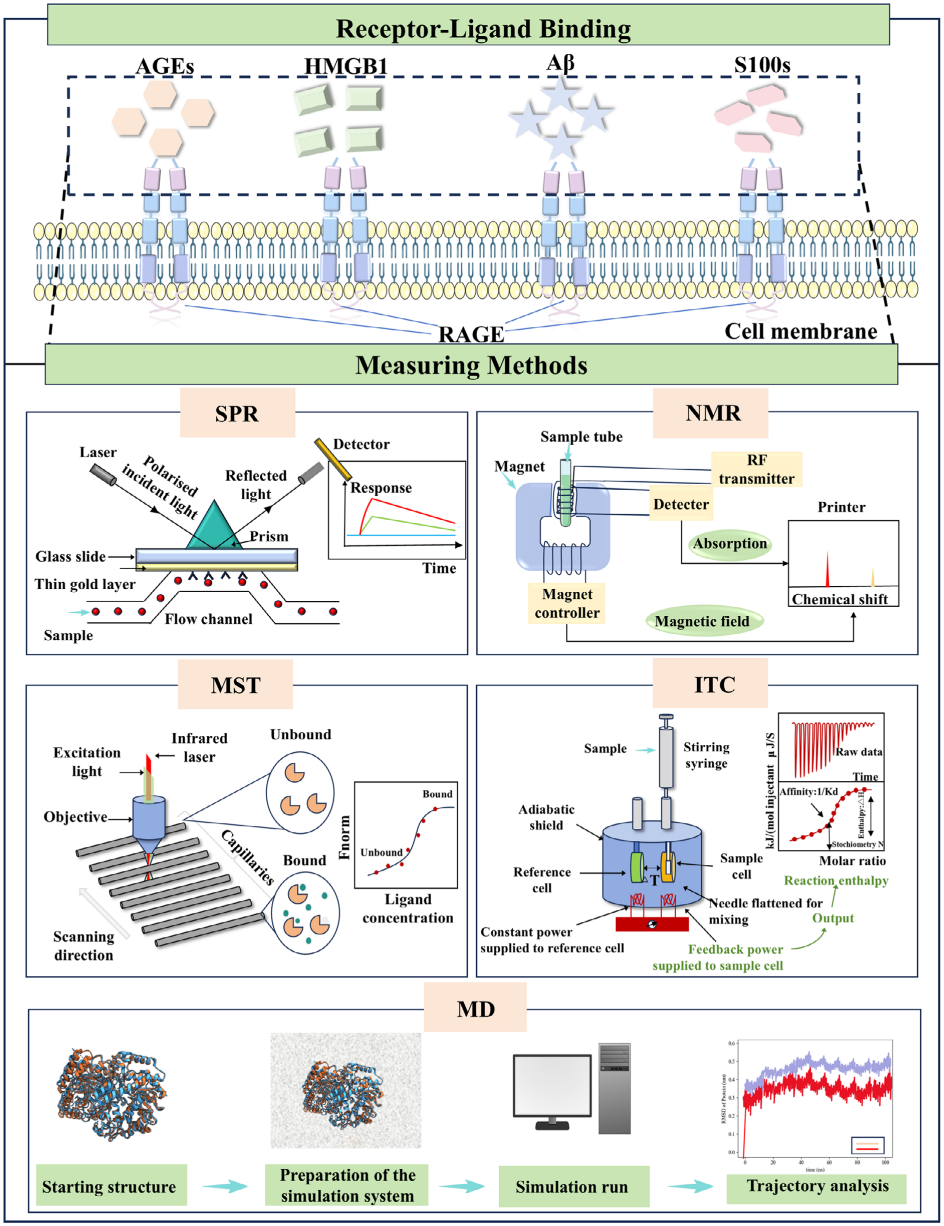
Methods of detecting binding kinetics between RAGE and its ligands. Commonly used methods to detect the binding kinetics of RAGE to its ligands are SPR, NMR, MST, ITC and MD simulations.

RAGE is a PRR with the ability to bind multiple ligands, the majority of which are oligomeric in nature.^[^ ^55^ ^]^ Initially identified as a receptor for AGEs, RAGE's activation state and ligand‐binding properties may undergo significant alterations with aging due to the continuous accumulation of AGEs. RAGE's multiligand capacity has been revealed through ongoing research.^[^ ^56^ ^]^ Its various isoforms enable it to bind a wide range of ligands, both endogenous and exogenous, including HMGB1, Aβ, endogenous and food‐derived AGEs, S100 proteins, Lysophosphatidic acid (LPA), phosphatidylserine (PS), complement protein C1q, IAPP, and others. Most RAGE ligands preferentially bind to the V‐type Ig structural domain or the V‐C1 Ig structural domain. In contrast, certain ligands, such as specific members of the S100/calcogranulin family, interact with the C‐type Ig structural domain of RAGE. In vitro studies have examined RAGE‐induced cellular responses using ligand‐induced activation and protein‐protein interaction assays. The binding of RAGE to its ligand triggers an immediate cellular response, a process that may be influenced by aging through alterations in cell membrane fluidity or changes in receptor glycation levels. Moreover, the intensity of the cellular signaling activated by RAGE‐ligand binding is influenced not only by the affinity between the interacting partners but also, more significantly, by the duration of the RAGE‐ligand signaling complex.^[^ ^55^ ^]^ Therefore, this paper focuses on the binding process of RAGE to its ligands, with particular attention to the binding and dissociation phases. The dissociation phase is especially significant, as it directly influences the duration of the signaling complex. This process may play a crucial role in the progression of chronic inflammatory and degenerative diseases associated with aging. Below, we present four commonly used methods for detecting the binding kinetics of the direct interaction between RAGE and its ligand (**Figure** **4**).

### 3.1 Binding Kinetics Between RAGE and Ligand

The signaling initiated by RAGE upon binding to its specific ligands is essential for various physiological processes, including proliferation, inflammation, apoptosis, angiogenesis, and chemotaxis. However, the balance of these signaling pathways may be disrupted with aging, leading to chronic inflammation and a diminished capacity for tissue repair. The harmful effects of RAGE are mediated through its interactions with ligands, and in aged individuals, these interactions may excessively activate downstream signaling pathways involving signal transducer and activator of transcription (STAT), c‐Jun N‐terminal kinase (JNK), and nuclear factor kappa‐B (NF‐κB). This overactivation is likely driven by the accumulation of chronic oxidative stress and an imbalance in immune homeostasis.^[^ ^57^ ^]^ Therefore, investigating the binding process of RAGE to its ligands may offer valuable insights into potential strategies for mitigating its harmful effects, particularly in the elderly population. Targeting the modulation of RAGE‐ligand interactions could represent a novel therapeutic approach for alleviating age‐related chronic diseases.

#### 3.1.1 AGEs

AGEs accumulate during aging, contributing to tissue damage and chronic inflammation, which in turn exacerbate the onset of aging‐related diseases such as cardiovascular disease, diabetes, and neurodegenerative disorders. AGEs accelerate the aging process by activating oxidative stress and inflammatory pathways through binding to their receptor, RAGE, ultimately disrupting cellular function. There are many types of AGEs, but not all interact with RAGE. The pattern and extent of glycation significantly influence structural and conformational changes, as well as the affinity for RAGE. Consequently, distinct and specific glycation patterns, particularly at specific residues, may be necessary for different target proteins to effectively recognize and activate RAGE.^[^ ^58^, ^59^ ^]^ One study compared the binding affinities of three glycosylated BSA ligands and found that glucose‐BSA and fructose‐BSA bind to RAGE more efficiently than the highly cross‐linked ribose‐BSA. The equilibrium dissociation constant (KD) values for these ligands with RAGE are 13 µM for glucose‐BSA and 19 µM for fructose‐BSA, indicating low‐affinity binding. These findings demonstrate that glycation‐mediated changes impact the structural properties of albumin and its binding capacity. Additionally, the study analyzed the binding efficiency of the glycated RAGE to different AGEs. Following glycation of RAGE, the binding efficiency decreased by a factor of 5.7 for glucose‐BSA, and no detectable binding was observed for fructose‐BSA. This indicates that glycosylated BSA has a low affinity for RAGE, and the binding affinity is influenced by the degree of protein cross‐linking.^[^ ^60^ ^]^ Molecular docking and molecular dynamics (MD) simulations revealed that carboxymethylation at specific lysine residues of human serum albumin (HSA), particularly Lys233 and Lys525, significantly influences its binding affinity to the V domain of RAGE. Among them, glycated HSA modified at Lys233 formed the greatest number of interprotein salt bridges and hydrogen bonds and exhibited the lowest binding energy, identifying it as the optimal binding site. In contrast, modification at Lys525 weakened direct electrostatic interactions with RAGE due to intramolecular salt bridge formation (Arg521‐Gln522), but induced a conformational change in HSA domain IIIB that enhanced hydrophobic embedding within RAGE, thereby maintaining strong binding stability. These findings suggest that specific glycation sites may represent novel therapeutic targets for disrupting RAGE‐mediated signaling.^[^ ^61^ ^]^

#### 3.1.2 HMGB1

HMGB1 is an important inflammatory mediator, and its concentration in the body generally increases with age. HMGB1 activates chronic inflammation and oxidative stress by binding to its receptor RAGE, processes that are closely linked to the onset and progression of various aging‐related diseases, including cardiovascular disease, diabetes mellitus, and AD. The HMGB1/RAGE axis has emerged as a pivotal factor in tumor growth and inflammation. Notably, HMGB1 interacts with RAGE in tumor cells but not in normal tissues.^[^ ^62^ ^]^ Research has shown that PARylated HMGB1 exhibits a higher affinity for PS and RAGE compared to its unmodified form.^[^ ^63^ ^]^ Additionally, KD for the interaction between HMGB1 and RAGE is 9.77 × 10^−8^ mol L^−1^, indicating that the binding affinity of HMGB1 to RAGE can be influenced by conformational changes in HMGB1.^[^ ^64^ ^]^

#### 3.1.3 S100 Protein

Research indicates that tetrameric S100B binds to sRAGE with greater affinity than dimeric S100B. Notably, a subset of tetrameric S100B (66%) demonstrates an eightfold higher binding affinity for sRAGE compared to its dimeric form. A second subset of tetrameric S100B (34%) exhibits ≈500‐fold greater binding affinity for sRAGE compared to dimeric S100B. Comparisons of kinetic parameters reveal that the dissociation of tetrameric S100B is 15 times slower than that of dimeric S100B. This slower dissociation of the ligand from the receptor leads to prolonged activation of the receptor‐dependent signaling pathway, leading to a stronger cellular response.^[^ ^65^ ^]^ S100A5 binds calcium ions and subsequently interacts with the V structural domain of RAGE to form a heterotrimer (KD ∼ 5.9 µM), a process that is unique among S100 family proteins. NMR titration experiments reveal chemical shift perturbation data indicating that S100A5 utilizes the periphery of the dimer interface to interact with the V domain of RAGE. The unique binding modes and stoichiometries of RAGE with various S100 family proteins may play a key role in modulating diverse RAGE signaling pathways.^[^ ^66^ ^]^

#### 3.1.4 Aβ

Aβ accumulates and forms plaques with age, playing a central role in AD. Aβ accumulation not only induces direct neurotoxicity but also triggers chronic inflammation and oxidative stress, which damage neurons and accelerate cognitive decline. This makes Aβ accumulation one of the key hallmarks and mechanisms of aging‐related neurodegenerative diseases. Aβ, as a ligand associated with RAGE, is transported across the blood‐brain barrier (BBB) in complexes with RAGE through transcytosis. Experimental data indicate that the binding constants for Aβ_40_ and Aβ_42_ are similar. For instance, RAGE expressed in Chinese hamster ovary cells binds Aβ_40_ with a constant of 75 ± 5 nM, while Aβ_42_ binds to RAGE on human neuroblastoma SH‐SY‐5Y cells with a constant of 92 ± 40 nM. RAGE interacts with soluble Aβ in the V‐domain region, exhibiting a binding constant of ≈52.2 ± 14.6 nM (Kd). The binding constant for Aβ_40_ with the V domain of RAGE is 1.6 × 10^6^ M^−1^ (Kd = 0.6 µM), which is lower than that for full‐length RAGE. In summary, Aβ forms stable complexes with RAGE, characterized by a high binding affinity. According to MD data, the native form of Aβ_42_ exhibits a strong interaction with RAGE, characterized by a binding constant of 6.95 × 10^12^ M^−1^. The data further indicate that both the pS8‐Aβ_42_ and isoD7‐Aβ_42_ isomers bind less efficiently than the native Aβ_42_. Notably, isoD7‐Aβ_42_ demonstrates the strongest binding to RAGE; docking studies revealed a greater number of contacts between RAGE and isoD7‐Aβ_42_ at Interface 1 compared to the native Aβ_42_. MD simulations indicated that the binding constant for the native form of Aβ is higher in the best complex configuration, despite having a smaller number of contacts.^[^ ^67^ ^]^ Furthermore, a significant difference exists between the binding affinities of Aβ for a single structural domain of RAGE and for the full‐length RAGE. Specifically, the binding constant of Aβ_1−40_ to the V domain of RAGE is ≈20 times smaller than that measured for full‐length RAGE (Kd = 57 nM). This suggests that the binding constants obtained for shorter Aβ variants with the V domain of RAGE may be considerably lower than those for full‐length RAGE.^[^ ^68^ ^]^

### 3.2 Detection Methods of Binding Kinetics

#### 3.2.1 Surface Plasmon Resonance (SPR)

SPR is a widely used method for detecting ligand‐receptor binding and dissociation processes. This technique is based on the principle that when a beam of light is directed onto a metal surface, free electrons in the metal interact with the photons, producing a fluctuation known as surface plasmon. Surface plasmons are electromagnetic waves that propagate near the surface of a metal. A resonance phenomenon, referred to as SPR, occurs when the propagation speed of the surface plasmon matches that of the incident light. In SPR, biomolecules (e.g., proteins, DNA) are adsorbed onto a metal surface, and the molecule of interest is introduced. The interaction between these biomolecules leads to a change in the resonance wavelength, which can be observed using the SPR phenomenon. This change is directly related to the interactions occurring between the biomolecules, allowing for the detection of binding and dissociation events. Consequently, SPR can be used to investigate the interaction of ligands with RAGE in detail, providing insights into the thermodynamics of binding (including stoichiometry and affinity) as well as the kinetics of binding and dissociation.^[^ ^55^ ^]^

Several research groups have utilized SPR to characterize various RAGE‐ligand interactions, revealing that multimeric ligands, in particular, exhibit prolonged binding times to the receptor. The S100 family of proteins has been extensively studied in vitro for their interactions with RAGE using SPR. A significant number of S100 proteins, including S100A1/A2, S100A4 through S100A9, S100A11/A12, S100B, and S100P, have been demonstrated to interact with RAGE in vitro. Moreover, cell‐based assays have demonstrated that all these S100 proteins, with the exception of S100A2 and S100A5, can trigger RAGE‐dependent signaling pathways. S100B predominantly interacts with the V domain of RAGE. Studies indicate that the interaction between dimeric S100B and the isolated V domain of RAGE (residues 23–132) exhibits a specific affinity in the submicromolar range, with a KD of ≈0.5 µM. S100A6 has been found to interact with both the V and C2 structural domains, while S100A12 can bind to the V and C1 structural domains of RAGE. Several S100 proteins, including S100B, S100A4, S100A8/A9, and S100A12, also interact with RAGE in their oligomeric state. Notably, the binding properties of these S100 proteins in their oligomeric forms differ from those of their corresponding dimers, indicating a more complex level of signaling modification and regulation.^[^ ^69^ ^]^ In the discovery or characterization of novel RAGE inhibitors, SPR can be employed to assess whether an inhibitor directly affects RAGE‐ligand binding. For instance, SPR analysis confirmed that one of the 4,6‐bis(4‐chlorophenyl)pyrimidine analogs can bind directly to RAGE, suggesting that this compound exerts its inhibitory effects by disrupting the interaction between RAGE and its ligands.^[^ ^70^ ^]^ Conversely, while heparin exhibits pro‐inflammatory activity by inhibiting HMGB1, SPR studies revealed that heparin does not interact directly with RAGE. Instead, it binds to HMGB1, thereby affecting the HMGB1‐RAGE affinity by altering the conformation of HMGB1.^[^ ^64^ ^]^ Furthermore, Xu et al.^[^ ^71^ ^]^ utilized a combination of Autodock, SPR, and DARTS analyses to demonstrate that tetrahydropyrogallolubin binds to the structural domains of RAGE. This interaction subsequently leads to the binding of PI1 to RAGE, resulting in the inactivation of the PI3K/AKT/NF‐κB pathway, thus identifying tetrahydropyrogallolubin as a potential RAGE inhibitor.

#### 3.2.2 Isothermal Titration Calorimetry (ITC)

ITC is a highly effective technique for studying protein interactions. It is the only truly label‐free method among the available techniques, measuring the heat absorbed or released during a reaction. This allows for direct access to the thermodynamics properties of the interaction. ITC can reliably quantify changes in binding constants and energies associated with the interaction of proteins with one another. Additionally, measurements obtained through ITC provide valuable information regarding the number of protein‐binding sites. Overall, ITC is a powerful tool for assessing the binding constants and energies of protein‐protein interactions.^[^ ^72^ ^]^

The ITC results for the mutant S100A6 bound to the V domain of RAGE revealed that the positively charged amino acids Arg48, Arg98, Arg104, Lys44, Lys107, Gly106, and Met102 contribute to a hydrophobic region. Additionally, the residues Gly47, Leu49, Glu50, Gln100, Asn103, and Asn105 interact with the mutant S100A6, positioning the basic amino acids close to the adjacent surface of the V domain of RAGE. This study showed that the C1 and C2 fragments, along with their associated linkers, configure a binding site for S100A6.^[^ ^73^ ^]^ Notably, the C3S mutant of S100A6 (S100A6m) exhibited a binding affinity of 3.00 µM for the V structural domain of RAGE. The positive values of both enthalpy and entropy suggest that the interaction between the two proteins is likely entropy‐driven, highlighting the significance of the buried nonpolar surface residues at or near the interface of the S100A6m‐V structural domain of the RAGE complex.^[^ ^74^ ^]^ ITC can also serve as a valuable tool for discovering novel inhibitors, such as single‐domain antibodies that specifically target the extracellular region of RAGE. The dissociation constants (Kd) for the V‐C1 structural domains interacting with three nanoantibodies (3CNB, 4BNB, and 5ENB) are determined to be 27.25, 39.37, and 47.85 nM, respectively. These results indicate that the binding affinity of the nanoantibodies for the V‐C1 structural domain is in the nanomolar range. Additionally, the blocking function of these nanobodies on the S100B‐V‐C1 structural domain was further evaluated using competitive ELISA. The findings suggest that these nanobodies possess significant therapeutic potential for RAGE‐related pathogenesis by inhibiting the interaction between S100B and RAGE.^[^ ^75^ ^]^

#### 3.2.3 Micro Thermophoresis Technology (MST)

MST is a powerful technique for studying protein‐protein and protein‐small molecule interactions. It provides valuable information on the binding dissociation between receptors and ligands, as well as insights into the interactions between receptors/ligands and small molecules, making it essential for the discovery and evaluation of potential small molecule inhibitors of RAGE.^[^ ^76^ ^]^ MST measures the equilibrium affinity constants of interactions by detecting changes in the sizes and charges of molecules, along with alterations in their hydration shells. Compared to other methods, MST has the advantage of avoiding surface immobilization and minimizing sample consumption. Moreover, it can be performed in a wide variety of buffers, including plasma and cell lysates.^[^ ^77^ ^]^

One study utilizing MST demonstrated that the binding affinity of geniposide to RAGE (Kd = 27.32 ± 11.54 µM) is significantly higher than that of AGEs to RAGE (Kd = 471.11 ± 135.63 µM). Notably, geniposide does not interact directly with AGEs. Molecular docking analyses revealed that geniposide can competitively block the binding of AGEs to RAGE by forming hydrogen bonds with residues N12, K15, W62, R66, E111, and E153 in the V domain of RAGE.^[^ ^78^ ^]^ Additionally, the Kd for Aβ_42_, the sRAGE containing phosphorylated Ser8 residues (pS8‐Aβ_42_), and the soluble extracellular fraction containing isoformylated Asp7 residues (iso‐Aβ_42_) with RAGE were determined using MST. These values were found to be 1.0 ± 0.2, 7 ± 2, and 23 ± 4 µM, respectively. Furthermore, the C‐terminal structural domain of Aβ_17‐42_ is shown to form a complex with sRAGE with a Kd of 10 ± 5 µM.^[^ ^79^ ^]^ MST and pulldown experiments also confirmed the binding of Prothrombin to sRAGE through its Gla structural domain, illustrating the interaction of prothrombin with the V‐C1 structural domain within the context of the entire extracellular domain. Interestingly, the affinity of sRAGE for prothrombin, as detected by MST, was found to be greater than that of the V‐C1 structural domain alone.^[^ ^80^ ^]^

#### 3.2.4 Nuclear Magnetic Resonance (NMR)

NMR is a powerful analytical tool that offers unique advantages over the previously discussed methods. Unlike those techniques, NMR not only provides kinetic characterization of interactions between proteins and other molecules, such as small‐molecule ligands, but also offers insights into the binding mechanisms of these proteins. Additionally, NMR can reveal structural changes that occur upon binding, enhancing our understanding of the dynamics of protein‐ligand interactions.

An experimental group conducted NMR spectroscopy on the crystal structure of the extracellular structural domains and the ligand‐binding domains of RAGE. Their findings revealed the presence of a hydrophobic cavity within the V structural domain of RAGE, characterized by a flexible region. This flexibility imparts plasticity to the hydrophobic cavity, enabling RAGE to interact efficiently with ligands. Notably, despite the structural diversity of AGEs, they exclusively bind to the V structural domain of RAGE.

Using NMR spectroscopy, it is determined that the V structural domain of RAGE is crucial for recognizing various types of AGEs. The study identified three distinct surfaces within the V structural domain that mediate interactions with AGEs, all located in the positively charged region. The first interaction surface comprises chain C and ring CC′, the second includes chain C′, chain F, and ring FG, while the third consists of chain A′ and ring EF. Notably, the secondary structural units of these interaction surfaces exhibit significant flexibility on the millisecond to microsecond timescales. Despite the specificity of the AGEs‐V structural domain interactions, the binding affinity of AGEs for the isolated V structural domain is relatively low, ≈10 µM.^[^ ^81^ ^]^

#### 3.2.5 MD Simulation

MD is a vital computational technique in the field of biomolecular systems, enabling the simulation and analysis of dynamic behaviors over time.^[^ ^82^ ^]^ MD simulations elucidate changes in biomolecular systems by tracing the dynamic binding processes between ligands and receptors. These simulations offer valuable structural insights into the energy landscape of ligand‐receptor complexes, playing a pivotal role in drug discovery and design.^[^ ^83^ ^]^ Through MD simulations, researchers can observe molecular motions, structural alterations, and the energetic features of interactions, which are critical for effective drug design. By constructing 3D structural models of RAGE receptors and specific ligands, MD simulations offer detailed information regarding energy changes, hydrogen bonding, van der Waals interactions, and other binding processes, all under physiological conditions. Additionally, when combined with free energy calculations, such as MM/PBSA or MM/GBSA methods, the binding affinity of ligands to RAGE can be quantitatively assessed. These methods can elucidate conformational changes, identify flexible regions, and evaluate the stability of both RAGE and its ligands upon binding. This provides a theoretical foundation for understanding RAGE‐mediated signaling in pathological processes and offers insights for the design of potential inhibitors or modulators. However, MD simulations require substantial computational resources, especially when long‐running simulations generate large datasets, which can present challenges for storage and analysis. To address these issues, high‐performance computing tools, such as MD‐Ligand‐Receptor Software, have been developed in recent years. These tools enable the parallelization of MD trajectory data, significantly enhancing analysis efficiency and providing a robust technological framework for the in‐depth study of RAGE‐ligand interactions and their binding dynamics.^[^ ^84^ ^]^

Structural studies play a crucial role in advancing our understanding of RAGE‐ligand interactions, providing deeper insights into the binding mechanisms involved. For instance, NMR spectroscopy and X‐ray crystallography analyses of RAGE's extracellular structural domains have demonstrated that ligands bind through various charge‐ and hydrophobic‐dependent mechanisms. In the specific case of Aβ binding to RAGE, it was observed that Aβ oligomers primarily interact with the V structural domain, while Aβ aggregates preferentially bind to the C1 structural domain. The binding affinity of soluble Aβ to the V structural domain is characterized by a dissociation constant of approximately Kd = 52.2 ± 14.6 nM. Notably, the major interaction region for Aβ within the V structural domain spans residues 17 to 23, which includes the strongly hydrophobic sequence 17‐LVFFA‐21, flanked by the negatively charged residues 22‐DE‐23 at the C‐terminal end. Overall, Aβ forms a stable complex with RAGE, exhibiting high binding constants and primarily engaging with the V and C1 structural domains.^[^ ^67^ ^]^

Most of the binding kinetics related to RAGE and its ligands have been investigated using various methods, as summarized in **Table** **2**. For in vitro studies, the initial step involves examining the binding kinetics between RAGE and its ligands or small molecule inhibitors. Once these interactions are established, further structural studies can be conducted to analyze the specific binding sites on RAGE. This sequential approach is significant for the targeted inhibition of RAGE and its ligands, as well as for the discovery of effective small‐molecule inhibitors.

**Table 2:** Information on the binding kinetics of RAGE to ligands.

Measurement Methods	Ligand	Interacting domain	Binding affinity(KD)/Dissociation constants(Kd)/Binding constants(Ka)	Refs.
**SPR**	AGEs (glucose‐BSA)	RAGE	KD = (1.39 ± 0.2) × 10^−5^ M	[60]
	AGEs (fructose‐BSA)	RAGE	KD = (1.95 ± 0.7) × 10^−5^ M	
	LPA	C2‐RAGE	KD = 6.35 × 10^−6^ M	[217]
		V‐RAGE	KD = 2.47 × 10^−9^ M	
	S100A8	RAGE	KD = 7.0 × 10^−8^ M	[218]
	S100A9	RAGE	KD = 3.209 × 10^−9^M	
	S100B (Dimer)	sRAGE	Kd = 8.3 × 10^−6^ M	[65]
	C1q	V‐RAGE	Kd = 5.6 × 10^−6^ M	[219]
	HMGB1	V‐RAGE	KD = 9.77 × 10^−8^ M	[64]
**ITC**	S100B	VC1‐RAGE	Kd = 3.19 ± 0.32 µM	[220]
	S100P	V‐RAGE	Kd = 6.2 ± 0.1 µM	[221]
	S100A13	C2‐RAGE	Kd = 1.30 µM	[38]
**MST**	AGEs	RAGE	Kd = 471.11 ± 135.63 µM	[78]
	Aβ_42_	V‐RAGE	Kd = 1.0 ± 0.2 µM	[79]
**NMR**	S100A11	V‐RAGE	Kd∼2.65 µM	[222]
**MD**	Aβ_42_	V/C1‐RAGE	Ka = 6.95 × 10^12^ M^−1^ Kd = 0.14 pM	[67]

## 4 RAGE Triggers Activation Pathways in Diseases of Aging

RAGE is a multi‐ligand PRR capable of binding a variety of structurally distinct ligands. It is expressed in many cell types and performs a wide range of functions. The ligand‐RAGE axis triggers a series of cellular signaling events that are associated with aging‐related diseases, including neurological disorders, inflammation, cancer, diabetes, and its complications, among others.^[^ ^22^ ^]^ Additionally, RAGE can mediate certain diseases in conjunction with other receptors. In this context, we will describe several well‐studied signaling pathways related to ligand‐RAGE binding.

### 4.1 Binding of RAGE to Ligands

**Figure 5:**
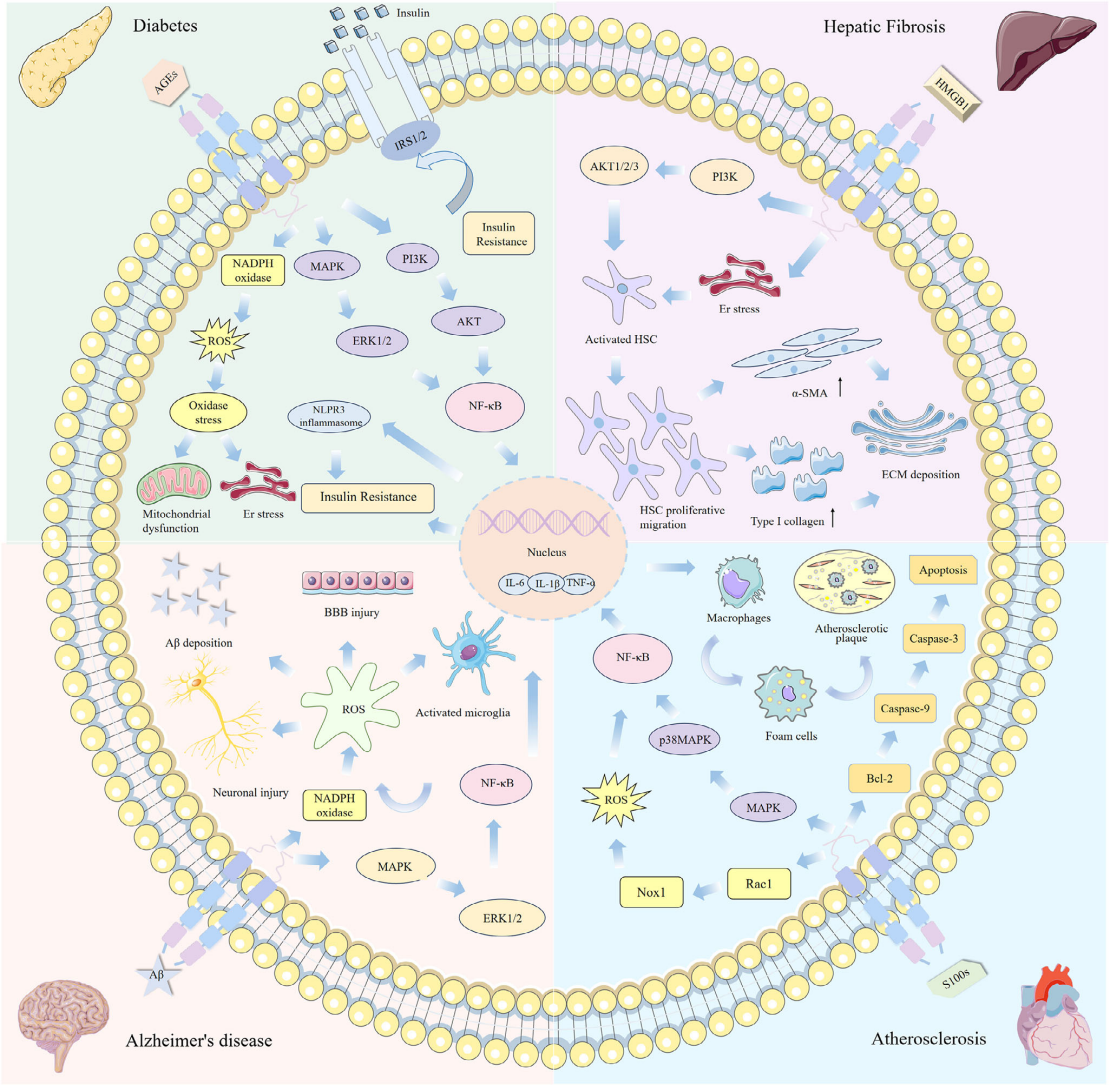
Triggers disease upon binding of RAGE to ligand.

The binding of RAGE to its ligands activates several cellular signaling cascades, including: NADPH oxidase, Mitogen‐Activated Protein Kinase Kinase/Extracellular Signal‐Regulated Kinase 1/2 (Ras/MEK/ERK1/2), Stress‐Activated Protein Kinase (SAPK)/JNK, Mitogen‐Activated Protein Kinase (MAPK)/p38, Phosphoinositide 3‐Kinase (PI3K)/AKT, Small GTPase Cdc42/Rac1, Janus Kinase (JAK)/STAT, Glycogen Synthase Kinase 3β (GSK‐3β). These pathways lead to the activation of various transcription factors, including NF‐κB, STAT3, Activator Protein 1 (AP‐1), and Early Growth Response‐1 (Egr‐1). This signaling cascade results in an increased synthesis and release of pro‐inflammatory cytokines such as interleukin (IL)‐1, IL‐6, and tumor necrosis factor alpha (TNF‐α) (**Figure** **5**). It has been demonstrated that these signaling pathways promote the continued production of RAGE, which in turn triggers the activation of additional pro‐inflammatory mediators. This creates a reinforcing feedback mechanism that amplifies the inflammatory reaction and contributes to disease progression.^[^ ^22^ ^]^ Additionally, ligand binding to the extracellular structural domains of RAGE is closely linked to the recruitment of DIAPH1 to the cytoplasmic structural domains, contributing to the generation of reactive oxygen species (ROS).^[^ ^85^ ^]^ The diversity of RAGE‐mediated cell signaling cascades adds to the complexity of disease development. Consequently, an effective strategy for preventing disease progression is to target the source by inhibiting RAGE activation of downstream signaling pathways. Furthermore, RAGE has the capacity to bind to multiple ligands, further complicating its downstream signaling. The following section explores the process of RAGE binding to ligands and its contribution to the development of various diseases.

#### 4.1.1 Diabetes Mellitus

Diabetes mellitus is a multifactorial metabolic disorder primarily characterized by hyperglycemia and is recognized as one of the most prevalent diseases of the 21st century. In particular, insulin sensitivity decreases with age, making blood glucose regulation more difficult and increasing the risk of developing diabetes. Metabolic changes commonly observed in the elderly, such as fat accumulation, chronic low‐grade inflammation, and oxidative stress, also contribute to the gradual decline in pancreatic islet function, further exacerbating the onset and progression of diabetes. Aging is a primary risk factor for major diseases such as cancer, cardiovascular disorders, and neurodegenerative conditions.^[^ ^86^ ^]^ At its core, aging results from the cumulative accumulation of biological damage across multiple levels, including telomere shortening, epigenetic dysregulation, mitochondrial dysfunction, and other hallmarks of cellular deterioration.^[^ ^87^ ^]^ Among these, excessive production of mitochondrial ROS is recognized as a critical initiating factor. In senescent cells, ROS levels can be several times higher than in young cells, leading to mitochondrial DNA mutations, protein oxidation, and lipid peroxidation.^[^ ^88^ ^]^ This persistent oxidative stress environment not only promotes cellular senescence but also triggers the senescence‐associated secretory phenotype (SASP),^[^ ^89^ ^]^ characterized by the release of pro‐inflammatory mediators such as IL‐6, IL‐8, and VEGF.^[^ ^90^ ^]^ These factors contribute to a state of chronic low‐grade inflammation, often referred to as “inflammaging.”^[^ ^91^, ^92^ ^]^ Importantly, oxidative stress and chronic inflammation form a self‐amplifying vicious cycle: inflammatory cytokines enhance ROS production via activation of the NF‐κB signaling pathway, which in turn promotes the accumulation of damage‐associated molecules such as AGEs, establishing a pathological basis for the activation of the RAGE signaling pathway.

Upon ligand binding, RAGE activates key pathways such as NF‐κB and MAPK, which in turn promote the expression of NADPH oxidase enzymes, amplifying oxidative stress and stimulating the release of SASP factors, including IL‐1β and CXCL1, thus intensifying inflammatory responses. This triad of oxidative damage, chronic inflammation, and RAGE activation forms a self‐reinforcing cycle that positions RAGE as a molecular switch commonly implicated in aging‐related pathologies. Despite the complex pathophysiology of diabetes, AGEs have been identified as a key factor in the progression of the disease and its associated complications.^[^ ^85^ ^]^ AGEs bind to their receptor, RAGE, triggering the induction of ROS through the activation of NADPH oxidases. This process contributes to further oxidative damage within cells, resulting in increased production of inflammatory cytokines.^[^ ^93^ ^]^ Furthermore, the RAGE‐AGE interaction enhances several signaling pathways, including PI3K, oncogenic Ras, Protein Kinase C (PKC), and Rho/GTPase partners (Cdc‐42 and Rac‐1). These pathways are essential for various cellular functions, including cell survival, stress responses, apoptosis, growth factor release, and cellular motility, all of which are critical in the context of inflammation and metabolic dysregulation.^[^ ^73^ ^]^ In diabetes, sustained hyperglycemia results in elevated levels of AGEs, which bind to their receptor, RAGE, activating multiple signaling pathways, including MAPK, JNK, ERK1/2, and JAK/STAT. These pathways result in the activation of transcription factors such as NF‐κB, STAT3, HIF‐1α, and AP‐1. Consequently, these transcription factors promote the expression of inflammatory cytokines, including IL‐1β, IL‐6, and TNF‐α, which exacerbate insulin resistance.^[^ ^94^ ^]^ Activation of JNK impairs insulin signaling by phosphorylating serine residues on insulin receptor substrate 1 (IRS‐1), while the sustained activation of NF‐κB creates a feedback loop that positively regulates RAGE expression.^[^ ^95^ ^]^ Additionally, RAGE/NF‐κB signaling contributes to insulin resistance through activating the NLRP3 inflammasome.^[^ ^96^ ^]^ Moreover, the interactions between AGEs and RAGE lead to increased production of ROS, triggering endoplasmic reticulum stress and impairing mitochondrial function, which further exacerbate oxidative stress and accelerate the inflammatory response.^[^ ^97^ ^]^ Ultimately, the interplay between ROS and the inflammatory response creates a vicious cycle that disrupts insulin signaling, resulting in decreased insulin sensitivity in diabetic patients.^[^ ^7^ ^]^

#### 4.1.2 Liver Fibrosis

Liver fibrosis is a reparative response of the liver to chronic injury, characterized by ECM. As people age, the liver's regenerative capacity declines, and its ability to repair and replace cells diminishes, making long‐term liver injury more likely to lead to liver fibrosis. Furthermore, metabolic disorders, chronic inflammation, and increased oxidative stress, which are prevalent in the elderly, further accelerate the progression of liver fibrosis. Activation of hepatic stellate cells (HSCs) is a crucial event in the progression of fibrosis, which is significantly influenced by the interaction between HMGB1 and RAGE. HMGB1, the first identified non‐AGE ligand for RAGE, plays a crucial role in liver pathology. Extracellular HMGB1 interacts with RAGE, Toll‐like receptors (TLRs), and cytoplasmic DNA/RNA sensor receptors, promoting inflammation through immune cell maturation, activation, and cytokine production.^[^ ^98^ ^]^ The binding of HMGB1 to RAGE activates several signaling pathways, including NF‐κB, MAPK, MEK, ERK1/2, PI3K, Akt, and transforming growth factor‐beta (TGF‐β).^[^ ^99^ ^]^ When released from damaged hepatocytes, HMGB1 binds to RAGE, activating HSCs and triggering their transdifferentiation into hepatic myofibroblasts. This process leads to the secretion of large amounts of type I collagen, contributing to fibrosis. Specifically, HMGB1 enhances collagen synthesis through the RAGE‐PI3K‐AKT and RAGE‐ERK signaling pathways.^[^ ^100^ ^]^ Studies have demonstrated a positive correlation between HMGB1 levels and HSC activation, proliferation, and migration. Moreover, HMGB1 induces HSC migration, stimulates the de novo synthesis of type I collagen and α‐smooth muscle actin, and promotes the production of ECM proteins, which accumulate in the liver, thereby accelerating the fibrosis process.^[^ ^101^ ^]^ Additionally, HMGB1 is implicated in inducing liver fibrosis through mechanisms involving endoplasmic reticulum stress.^[^ ^102^ ^]^

#### 4.1.3 Atherosclerosis

Atherosclerosis is a blood vessel disorder characterized by abnormal arterial calcification, oxidative stress, and dyslipidemia. As people age, endothelial function in blood vessels gradually declines, leading to reduced arterial elasticity and thickening of the vessel walls. Additionally, chronic inflammation, oxidative stress, and lipid metabolism disorders, which are common in the elderly, contribute to the development and progression of atherosclerosis, increasing the risk of cardiovascular disease. The core mechanism underlying aging‐associated atherosclerosis involves a self‐perpetuating cycle of chronic vascular inflammation and dysregulated lipid metabolism. With advancing age, endothelial dysfunction, oxidative stress, and disruption of lipid homeostasis collectively drive atherosclerotic progression.^[^ ^103^ ^]^ On one hand, age‐related mitochondrial decline and dysregulation of nuclear receptor signaling pathways, particularly peroxisome proliferator‐activated receptor alpha (PPARα) and liver X receptor, impair fatty acid oxidation and reverse cholesterol transport, leading to lipid accumulation within the vascular wall.^[^ ^104^ ^]^ On the other hand, SASP contributes to persistent inflammation through the release of cytokines such as IL‐6 and TNF‐α, which activate the sterol regulatory element‐binding protein 1c pathway. This, in turn, increases the production of pro‐atherogenic lipoproteins. The result is a vicious cycle of “lipotoxicity‐vascular injury” that perpetuates atherogenesis in the aging vasculature.^[^ ^105^ ^]^

Among the various factors implicated in this condition, the S100 family of calcium‐binding proteins stands out. Over 20 members of the S100 family have been identified so far, with S100A8, S100A9, and S100A12 being strongly linked to cardiovascular disease.^[^ ^106^ ^]^ Some members of the S100 protein family are secreted extracellularly in an autocrine or paracrine manner, where they can trigger various inflammatory signaling pathways. They achieve this through interactions with a range of cell surface receptors, such as RAGE, TLR4, GPCR, and scavenger receptors. These interactions eventually activate pro‐inflammatory transcription, resulting in the production of ROS and apoptotic cell death.^[^ ^107^ ^]^ Current findings indicate that RAGE is the primary binding receptor for S100 proteins, with S100A12 identified as the strongest RAGE‐activating factor within this family.^[^ ^108^ ^]^ Released from monocytes, smooth muscle cells, and endothelial cells in response to cellular stress stimuli, S100A12 binds to RAGE and initiates a cascade of signaling cascade reactions. This process activates the MAPK/ERK signaling pathway, promotes intracellular phosphorylation of ERK1/2, activates the transcription factor AP‐1, and induces the expression of pro‐inflammatory genes.^[^ ^109^ ^]^ Furthermore, S100A12 activates the NF‐κB signaling pathway via RAGE, prompting the translocation of NF‐κB to the nucleus and initiating the transcription of pro‐inflammatory cytokines, such as TNF‐α, IL‐6, and IL‐1β.^[^ ^110^ ^]^ The synergistic effects of these signaling pathways further aggravate endothelial cell dysfunction and vascular inflammation, driving the progression of atherosclerosis, promoting plaque formation, and contributing to vascular injury. Moreover, S100A8/A9 promotes chemotaxis of macrophages and neutrophils through the RAGE pathway, enhancing intravascular inflammatory responses and further driving plaque formation and the progression of atherosclerosis. Notably, S100A8/A9 can also bind to scavenger receptors (e.g., cluster of differentiation 36) and TLR4 through RAGE‐independent pathways, activating inflammatory cells. Thus, S100 proteins are key pro‐inflammatory factors in the development and progression of atherosclerosis by activating RAGE and other inflammatory pathways.^[^ ^111^ ^]^ The RAGE‐S100A12 axis functions as a central molecular hub in aging‐related atherosclerosis. The marked accumulation of S100A12 in aged vascular tissue activates the NF‐κB and p38 MAPK signaling pathways via high‐affinity binding to the RAGE receptor. This activation exerts multiple pathological effects: it may suppress PPARα‐CPT1A‐mediated fatty acid oxidation, thereby promoting lipid accumulation;^[^ ^112^ ^]^ it can also induce ROS bursts that facilitate the oxidative modification of low‐density lipoprotein, accelerating foam cell formation.^[^ ^113^ ^]^ Additionally, this signaling may trigger premature endothelial cell death and increase vascular permeability.^[^ ^114^ ^]^ Notably, RAGE expression is progressively upregulated with age, forming a self‐amplifying feedback loop with S100A12, an “activation‐re‐expression” cycle that intensifies lipid metabolic disruption and vascular inflammation, ultimately contributing to plaque instability. Therapeutically targeting the RAGE‐S100A12 axis holds promise as a dual‐intervention strategy, simultaneously addressing dyslipidemia and chronic inflammation. As such, it represents a compelling approach for the treatment of aging‐associated atherosclerosis.

#### 4.1.4 AD

AD is a progressive and multifaceted neurodegenerative disorder. Aging is a major risk factor for AD, with neurodegenerative changes in the brain becoming more pronounced over time, particularly the accumulation of Aβ and the abnormal phosphorylation of tau proteins. These pathological changes result in neuronal damage, synaptic loss, and brain atrophy, all of which impair cognitive function and increase the risk of developing AD. Aβ, a cleavage product of the amyloid precursor protein (APP), is generated through the hydrolytic cleavage of proteins and serves as the primary neuropathological marker of AD.^[^ ^115^ ^]^ BBB is a critical structure that maintains the stability of the brain's internal environment, preventing the unrestricted entry of peripheral circulating substances into the brain. In the context of AD, RAGE is upregulated and interacts with Aβ, contributing to the neurotoxic effects of Aβ within the brain. Aβ activation of RAGE induces oxidative stress in neurons and stimulates the production of pro‐inflammatory cytokines in microglial cells by activating NADPH oxidase and increasing the generation of ROS.^[^ ^22^, ^73^ ^]^ Moreover, studies have shown that the interaction between RAGE and Aβ expressed on brain endothelial cells activates MAPK, JNK, and ERK. This activation of these signaling pathways promotes the production of endothelial MMP‐2, which is linked to the vascular inflammatory responses observed in AD. There is evidence that the activation of microglia via RAGE‐Aβ interactions also involves the p38 MAPK signaling pathway. Additionally, some researchers have demonstrated that the overexpression of RAGE in microglia leads to increased production of pro‐inflammatory mediators, such as IL‐1β and TNF‐α, following Aβ stimulation in a transgenic animal model of AD. This heightened cytokine production was associated with elevated levels of phosphorylated p38 and ERK1/2.^[^ ^116^ ^]^ The resultant pro‐inflammatory cytokines disrupt BBB, promoting neuronal apoptosis and exacerbating inflammatory responses.^[^ ^117^ ^]^

### 4.2 Possible Co‐Actions of RAGE with Other Receptors

The mechanisms underlying disease in the human body are diverse, and some conditions may not arise from the activation of a single receptor but rather from the combined actions of multiple receptors. RAGE can interact with other cell surface receptors to initiate specific signaling pathways. For example, nerve growth factor oligomerizes upon glycation, and RAGE co‐signals with the p75 neurotrophin receptor to induce motor neuron death at low physiological concentrations.^[^ ^118^ ^]^ Additionally, several receptors co‐ligand with RAGE, including AGEs that also bind to fasciclin, galectin‐3,^[^ ^119^ ^]^ EGF‐like proteins, laminin‐type EGF‐like proteins, and scavenger receptor‐1.^[^ ^120^ ^]^ Other receptors, such as cluster of differentiation 36,^[^ ^121^, ^122^ ^]^ scavenger receptor class A, and lectin‐type oxidized low‐density lipoprotein receptor 1, also interact with RAGE. Furthermore, Aβ and HMGB1 bind to TLRs,^[^ ^123^ ^]^ while S100A8/A9 interacts with Emmprin. However, Emmprin and TLR2/4 do not directly bind to RAGE.^[^ ^124^ ^]^ The signaling mechanisms associated with RAGE in these processes are quite complex.^[^ ^125^ ^]^ HMGB1 activates the RAGE signaling pathway, which involves Cdc42/Rac and MAPKs, ultimately leading to NF‐κB‐dependent transcriptional activity. Additionally, HMGB1 can activate TLR pathways, specifically TLR2 and TLR4, which also signal toward NF‐κB. TLRs can activate NF‐κB through the MyD88, IRAK, and TRAF signaling intermediates, as well as through Rac1, PI3K, ERK1/2, and p38 MAPK. RAGE similarly activates Rac1, Cdc42, Ras, and p38 MAPK, which together form two distinct signaling pathways that converge on NF‐κB to induce gene expression.^[^ ^126^ ^]^

**Figure 6:**
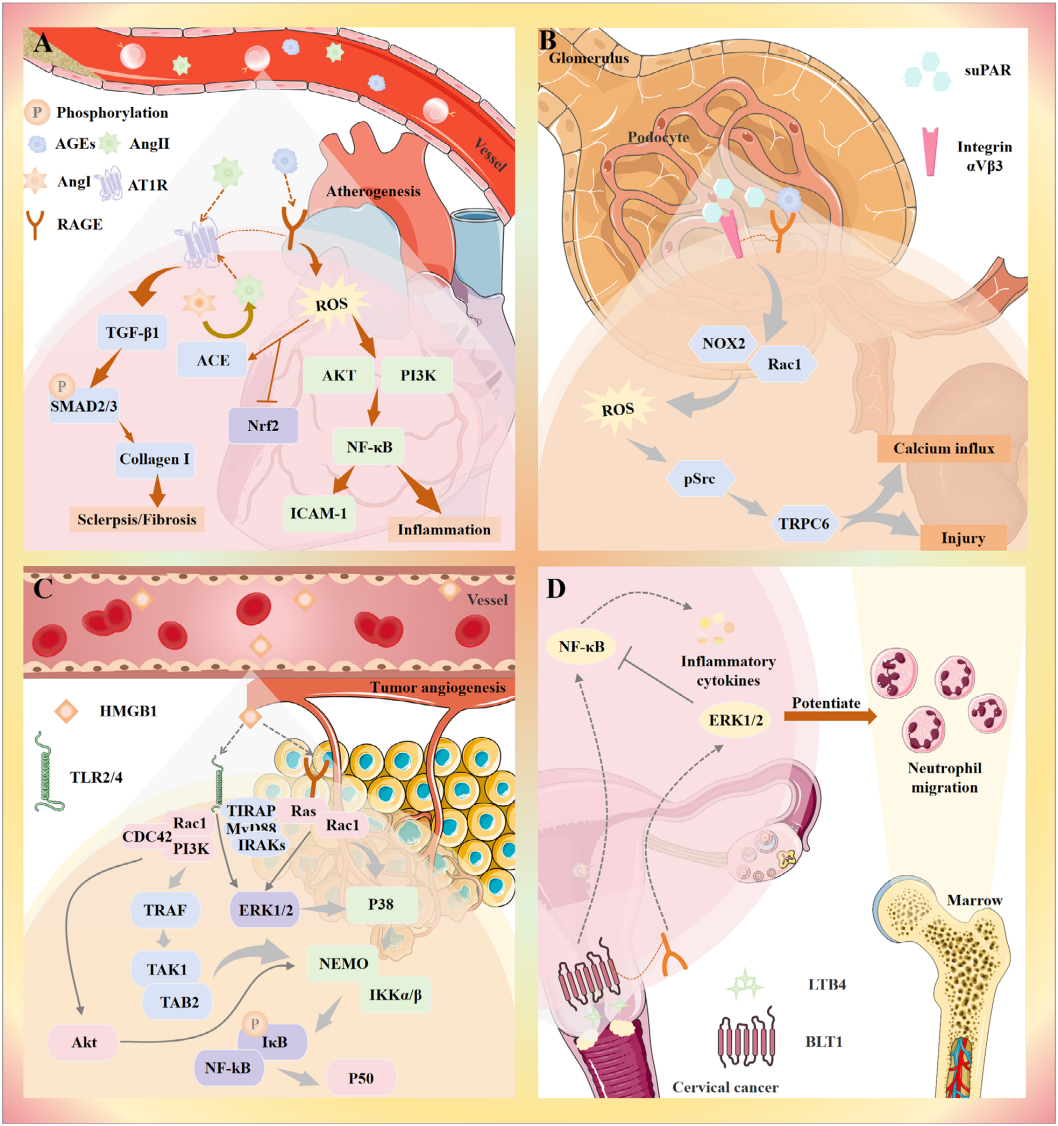
Cell signaling pathways in which RAGE acts cooperatively with other receptors. A) Signaling pathways co‐mediated by RAGE and AT1R. B) Signaling pathways co‐mediated by RAGE and αVβ3 integrins. C) Signaling pathways co‐mediated by RAGE and TLR2/4. D) Signaling pathways co‐mediated by RAGE and BLT1.

RAGE can form complexes with other receptors to stimulate signaling pathways. In podocytes, for example, RAGE associates with αVβ3 integrins to regulate TRPC6 activity.^[^ ^127^ ^]^ The binding of AGEs or soluble urokinase plasminogen activator receptor (suPAR) activates NADPH oxidase 2 (NOX2) and Rac1, resulting in the production of ROS. These ROS, in turn, activate Src kinases, leading to an increased expression of the calcium channel TRPC6; alterations in TRPC6 activity are associated with podocyte injury.^[^ ^128^ ^]^ Additionally, research has shown that the type 1 angiotensin II receptor (AT1R) can form a heterodimerization complex with RAGE. The angiotensin (Ang) II‐induced activation of AT1R transactivates RAGE and promotes NF‐κB‐driven pro‐inflammatory gene expression.^[^ ^129^ ^]^ In mouse thylakoid membrane cells, AGEs enhance RAGE expression, which induces ROS production.^[^ ^130^ ^]^ This increase in ROS may potentiate the activation of the downstream PI3K/AKT pathway, decrease the production of Nrf2, and subsequently lead to NF‐κB activation and pro‐inflammatory cytokine production.^[^ ^127^, ^131^ ^]^ Additionally, BLT1 (leukotriene B4 receptor 1) has been identified as a novel co‐receptor for RAGE. The findings from their study clearly demonstrate that RAGE binding modulates BLT1 signaling in both positive and negative manners by regulating the key effector kinase, ERK1/2.^[^ ^132^ ^]^ The signaling of the LTB4 ligand‐BLT1 is influenced by RAGE in these dual capacities. Specifically, the alternately activated ERK1/2 inhibits the LTB4‐BLT1‐mediated activation of NF‐κB, thereby reducing the production of inflammatory cytokines, while simultaneously enhancing the LTB4‐BLT1‐mediated induction of chemotaxis(**Figure** **6**).^[^ ^133^ ^]^ In the context of cardiomyopathy, the β1‐adrenergic receptor (β1AR) and RAGE exhibit interdependence, physically interacting to form a protein complex. This complex triggers cardiomyocyte death, myocardial remodeling, and heart failure through the activation of CaMKII.^[^ ^134^ ^]^ Furthermore, Aβ1‐42 has been shown to bind to formyl peptide receptors, which may interact with RAGE and enhance ERK1/2 signaling in HEK293 cells.^[^ ^135^ ^]^

## 5 Dietary Active Ingredients Modulate Diseases of Aging via RAGE

Dietary contains a wide variety of bioactive components, including terpenoids, flavonoids, alkaloids, steroids, lignans, and minerals. Many of these substances exhibit physiological benefits for the human body and have been shown to possess anti‐inflammatory, anti‐tumor, and anti‐glycation properties.^[^ ^136^, ^137^, ^138^ ^]^ Consequently, the physiological functions of these dietary components may be linked to the inhibition of RAGE. Most of these bioactive compounds either contribute to the ligand‐generating process for RAGE or influence the activation of downstream cell signaling pathways mediated by RAGE. While many active ingredients in food participate in these processes, some studies have also reported direct interactions with RAGE that inhibit ligand‐RAGE interactions (**Table** **3**). Here, we will briefly discuss three common dietary active ingredients, polyphenols, polysaccharides, and terpenoids, and their pathways that inhibit RAGE‐induced diseases associated with aging.^[^ ^139^ ^]^

**Table 3:** Active components in food inhibiting RAGE‐induced diseases and their inhibitory mechanisms.

Category	Source	Name	Main components	Inhibitory mechanism	Refs.
**Polyphenols**	Rheum palmatum L.	4′‐Methoxyresveratrol	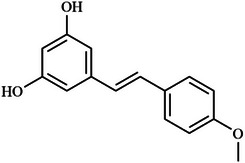	Inhibiting NLRP3 inflammasome activation and RAGE‐mediated MAPK/NF‐κB signaling pathway.	[143]
	Cornus officinalis Sieb. et Zucc.	7‐O‐Galloyl‐d‐sedoheptulose	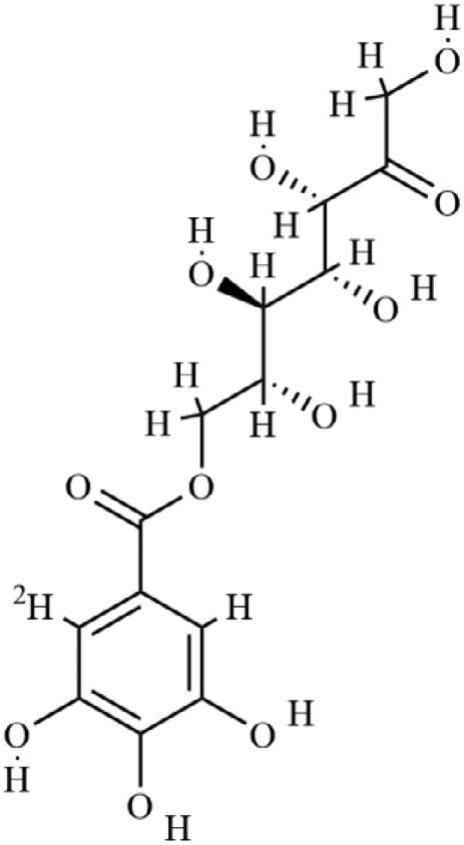	Reducing the increase in fluorescent AGEs and decreasing the expression of RAGE and AGE‐related proteins.	[223]
	Apple tree and apple pericarp	Phloretin	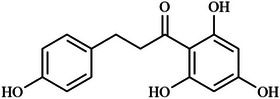	Preventing the formation of AGEs and inhibiting the RAGE/p38 MAPK/NF‐κB signaling pathway.	[224]
	Celery, green pepper, and chamomile	Luteolin	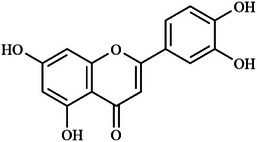	Upregulation of hippocampal SIRT1 mRNA expression and modulation of the GLO1/AGEs/RAGE axis.	[225]
	Thunbergia erecta leaf	Rosmarinic acid	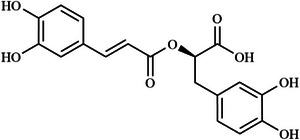	Suppressing the expression of HMBG1, RAGE, p65 (NF‐kB), and IL‐1β.	[226]
	Wein und Traubensaft	Resveratrol	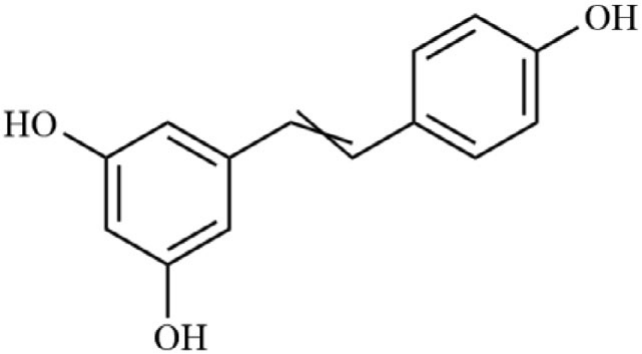	Inhibiting the AGE‐RAGE and HIF‐1 signaling pathways.	[147]
	Black tea, etc.	Theaflavins	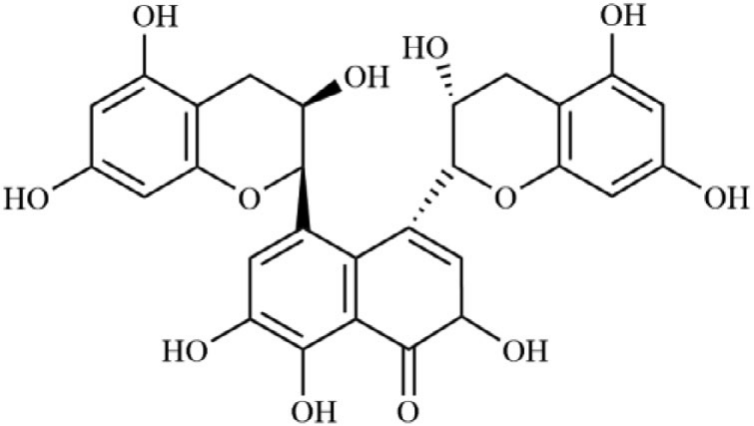	Inhibiting the formation of AGEs, thereby inhibiting the activation of the MAPK/NF‐κB signaling pathway	[227]
**Polysaccharides**	Fagopyrum esculentum	F. esculentum polysaccharides	Rhamnose, glucose and galactose (1:67.4:1.9)	Mitigating oxidative stress, RAGE/p38/NF‐κB‐mediated neuroinflammation, and AD‐associated proteins by upregulating autophagy and SCFA levels.	[155]
	Auricularia auricula	Auricularia auricula polysaccharide	Fructose, rhamnose, arabinose, galactose, glucose and mannose (20.3:11.8:14.6:28.1:10.7:14.5)	Modulating the RAGE/TGF‐β/NOX4 pathway.	[228]
	Astragali radix	Astragalus polysaccharides	Arabinose, glucose, galacturonic acid and glucuronic acid	Inhibiting the HMGB1/RAGE/NF‐κB/NLRP3 signaling pathway.	[229]
	Fruits of Phyllanthus emblica L.	Phyllanthus emblica L. polysaccharides	Mannose, rhamnose, galacturonic acid, glucose, galactose, arabinose, and fucose (1.2:1.0:2.8:2.3:8.7:1.7:3.0)	Downregulating the activation of the RAGE/NF‐κB and MAPK pathways by suppressing the abundance of inflammatory‐related bacteria while promoting the growth of SCFA.	[156]
	I. obliquus	Inonotus obliquus polysaccharide	Glucose, galactose, xylose, mannose and arabinose (29.094:21.705:14.857:9.375:7.709)	Inhibiting the final formation of AGEs.	[230]
	Dry roots of Angelica sinensis	Polysaccharides from Angelica sinensis	Rhamnose, glucose, galactose, and arabinose (1.0:6.9:4.1:2.3)	High affinity for RAGE, and modulating the RAGE‐JNK/p38‐IRS signaling pathway.	[153]
	Dry root bark of perennial peony of the Paeonia genus	Polysaccharide from Moutan Cortex	D‐glucose and L‐arabinose (3.31:2.25)	Decreasing significantly the serum levels of AGEs and RAGE.	[231]
**Terpenoids**	Ginkgo biloba leaf	Ginkgolide B	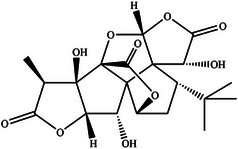	Reducing the levels of RAGE.	[232]
	Agastache rugosa	β‐Caryophyllene	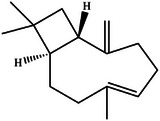	Downregulating the RAGE signaling pathway.	[163]
	The root of licorice	Glycyrrhizin	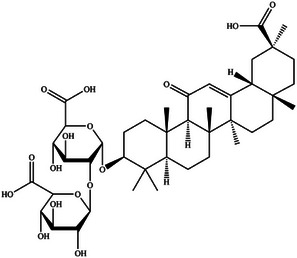	Regulating the HMGB1‐RAGE‐NF‐kB/AKT signalling pathway	[159]
	Root of herbaceous peony (Paeonia lactiflora)	Paeoniflorin	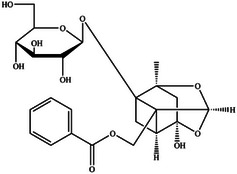	Blocking RAGE mRNA upregulation.	[233]
	Fruits of Cornus Officinalis Sieb. Et Zucc	Cornuside	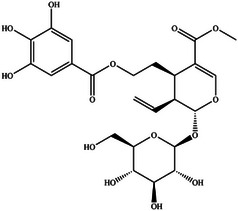	Binding to the RAGE, directly impeding the interaction of Aβ and RAGE	[234]

### 5.1 Dietary Polyphenols

Polyphenols are a varied class of compounds present in various foods. Major sources of polyphenols such as fruits, vegetables, legumes, tea, and red wine.^[^ ^140^ ^]^ The most prevalent categories of polyphenols are flavonoids, phenolic acids, lignans, and stilbenes. Numerous studies have verified that different polyphenols possess anti‐glycation properties, both in vivo and in vitro.^[^ ^141^, ^142^ ^]^

4′‐Methoxyresveratrol (4′Mr), also known as 3,5‐dihydroxy‐4′‐methoxystilbene, belongs to the stilbene group derived from the plant families Dipterocarpaceae and Chrysomelidae, which have limited information regarding their biological activities. Concurrent treatment with 4′Mr, a stilbene similar to resveratrol, effectively reversed trans‐overexpression. Furthermore, 4′Mr significantly inhibited the expression of oxidative stress and inflammatory biomarkers induced by AGEs. These findings suggest that 4′Mr may play a role in the RAGE receptor‐ligand axis by suppressing RAGE expression. Specifically, 4′Mr inhibited ROS production by down‐regulating both RAGE and NOX expression. The anti‐inflammatory effects of 4′Mr are primarily mediated by blocking the RAGE‐mediated signaling pathway interactions with MAPKs and nuclear factor NF‐κB, as well as inhibiting NLRP3 inflammasome activation.^[^ ^143^ ^]^

Epigallocatechin gallate (EGCG), along with rhizopodophyllin, 6‐sagarol, and curcumin derived from apple, demonstrated a protective effect on methylglyoxal (MGO)‐treated human renal proximal epithelial cells. Inhibition of RAGE expression significantly reduced MGO‐induced carbonyl stress. Consequently, the function of Nrf2 was activated, resulting in its translocation from the cytoplasm to the nucleus, which in turn induced the levels of phase II detoxification enzymes, such as heme oxygenase‐1 (HO‐1). EGCG notably decreased the expression of the pro‐inflammatory cytokine TNF‐α, along with the mRNA and protein expression of RAGE. The cytoprotective mechanisms involved are primarily associated with the regulation of RAGE expression, the modulation of MAPK and TGF‐β pathways. Specific regulatory actions include the activation of Nrf2 and the enhanced expression of HO‐1, a crucial detoxifying enzyme that protects against ROS within the KEAP1‐Nrf2‐antioxidant response element pathway. Additionally, there is down‐regulation of upstream regulators, such as nuclear factor NF‐κB and IκBα, along with corresponding decreases in downstream inflammatory enzymes, including inducible nitric oxide synthase (iNOS), cyclooxygenase‐2 (COX‐2), IL‐6, TNF‐α, and MMPs.

Tea and its polyphenolic compounds are essential in regulating the expression of RAGE, particularly through MAPK and TGF‐β pathways. Tea polyphenols effectively promote the KEAP1‐Nrf2 pathway while inhibiting the MAPK and TGF‐β pathways, thereby alleviating conditions such as nephropathy, vasculopathy, proliferation, apoptosis, inflammation, and angiogenesis. Both quercetin and tannic acid have been shown to inhibit the formation of RAGE ligands. Quercetin specifically disrupts Aβ biogenesis by influencing APP processing through the inhibition of β‐secretase (BACE1) activity.^[^ ^144^ ^]^ In the context of AD, tannic acid also affects the handling of APP by inhibiting BACE1 activity and preventing the cleavage at the β‐site. Both polyphenols ultimately result in a decrease in Aβ levels, a ligand for RAGE.^[^ ^145^ ^]^ Resveratrol is a non‐flavonoid polyphenol known for its efficacy in treating high blood pressure, cardiovascular diseases, and even pre‐eclampsia. It is naturally found in foods such as grapes, red wine, berries, and peanut skins.^[^ ^146^ ^]^ Resveratrol exhibits strong binding affinity to key inflammatory and growth factors, including IL‐6, TNF, IL‐1β, vascular endothelial growth factor A, and the epidermal growth factor receptor. Its therapeutic effects are primarily mediated through the modulation of the AGEs‐RAGE signaling pathway.^[^ ^147^ ^]^ At the molecular level, resveratrol inhibits AGE–RAGE interactions by competitively occupying the hydrophobic ligand‐binding pocket within the V‐domain of RAGE. It forms hydrogen bonds with Leu79 and Pro80, as well as hydrophobic interactions with Val78 and Gln67, residues essential for receptor oligomerization and ligand engagement. This steric hindrance effectively blocks the binding of pathogenic AGEs and S100 proteins, as confirmed by stable complex formation observed in 100‐ns MD simulations (RMSD ≈ 0.5 nm).^[^ ^148^ ^]^ In addition, resveratrol directly targets multiple downstream kinases involved in RAGE signaling, including NF‐κB, PI3K, PKC, and ERK, through high‐affinity interactions at their catalytic sites (e.g., PI3K ATP‐binding pocket: −8.6 kcal mol^−1^; ERK conserved Lys54). Functionally, it suppresses AGE‐induced proliferation in colorectal cancer cells, with an IC_50_ of 110 µM against methylglyoxal.^[^ ^148^ ^]^ In general, these findings highlight resveratrol's dual precision mechanism: 1) competitive antagonism of RAGE and 2) direct inhibition of key signaling kinases at atomic resolution.

### 5.2 Dietary Polysaccharides

Polysaccharides are natural polymer compounds formed through the condensation of more than ten monosaccharide molecules. Dietary polysaccharides primarily originate from edible plants, animals, microorganisms, and novel food materials. Among these, plant‐derived polysaccharides represent a major natural source and exhibit significant cancer‐preventive properties, making them valuable for applications in the food and pharmaceutical industries. These polysaccharides are predominantly found in grains, legumes, potatoes, fruits, vegetables, and algae. Additionally, many medicinal plants, such as Angelica sinensis and Astragalus membranaceus, provide dietary polysaccharides with important biological activities. Beyond their fundamental nutritional value, these polysaccharides exhibit diverse biological functions, including immune regulation, antioxidant activity, hypoglycemic effects, and antibacterial and anti‐inflammatory properties.^[^ ^149^, ^150^ ^]^ Due to their excellent bioactivity and safety, polysaccharides derived from medicinal plants and food sources hold significant potential for the development of functional foods and pharmaceuticals. In particular, medicinal and edible resources such as Angelica sinensis, Astragalus, buckwheat, and cloves have garnered considerable research interest due to their potent biological activities, low cytotoxicity, and overall safety.^[^ ^151^ ^]^ The structural properties of these polysaccharides are influenced by factors such as molecular weight, monosaccharide composition, branching characteristics, and modification potential.^[^ ^152^ ^]^

Polysaccharides are known for their diverse biological activities, typically exhibiting gentle yet effective actions. Some glycosaminoglycans have been reported to inhibit RAGE activation. Specific polysaccharides from Angelica sinensis, particularly APS‐1I, exhibit high‐affinity binding to RAGE, as quantified by MicroScale Thermophoresis (Kd = 2.02 ± 0.2 µM). This binding affinity is markedly stronger than that of the simpler linear glucan APS‐2II (Kd = 85.92 ± 0.2 µM) and the arabinoglucan APS‐1d. The superior interaction of APS‐1I is attributed to its complex heteropolysaccharide architecture, featuring a backbone composed of α‐1,6‐Glcp, α‐1,3,6‐Glcp, α‐1,2‐Glcp, α‐1,4‐Galp, and α‐1,3‐Rhap residues, along with extensive branching enriched in arabinofuranose motifs (α‐T‐Araf, α‐1,3‐Araf, α‐1,3,5‐Araf). Although α‐1,6‐Glcp linkages are present across all three polysaccharides, high‐affinity RAGE binding requires their co‐occurrence with complex branched structures and is independent of molecular weight (APS‐1I: 17.0 kDa; APS‐1d: 5.1 kDa; APS‐2II: 10.0 kDa). Mechanistically, APS‐1I competes with pathogenic ligands (e.g., AGEs) for RAGE binding, thereby downregulating RAGE expression and inhibiting downstream phosphorylation of JNK and p38 MAPK in insulin‐resistant cells and diabetic models. This inhibition reduces the phosphorylation of insulin receptor substrates (IRS‐1 at Ser307 and IRS‐2 at Ser731), facilitating the restoration of insulin signaling. Notably, siRNA‐mediated RAGE knockdown abolishes APS‐1I's effect on JNK/p38 phosphorylation, confirming RAGE as its direct molecular target. Collectively, these findings underscore that the unique, arabinofuranose‐rich branched architecture of APS‐1I underlies its specific, high‐affinity interaction with RAGE and its therapeutic modulation of the RAGE–JNK/p38–IRS signaling axis, contributing to its potent hypoglycemic effects.^[^ ^153^ ^]^

Research has also demonstrated that Liriope spicata var. prolifera, which is primarily composed of fructose and glucose, effectively reduces the levels of AGEs in diabetic rats. This reduction is associated with the down‐regulation of the AGE‐RAGE system, thereby preventing the onset of diabetic nephropathy.^[^ ^154^ ^]^ Additionally, polysaccharides isolated from Fagopyrum esculentum have been demonstrated to inhibit neuroinflammation as well as the expression of BACE1 and Aβ proteins through the RAGE/p38/NF‐κB signaling pathway. These results imply that such polysaccharides hold potential for limiting aberrant APP‐Aβ metabolism and may, therefore, help prevent the progression of AD.^[^ ^155^ ^]^

Astragalus polysaccharides, specifically AP1 and AP2, have demonstrated protective effects on astrocytes against oxygen‐glucose deprivation/reperfusion‐induced neuroinflammation by blocking the HMGB1/RAGE/NF‐κB/NLRP3 signaling pathway. Additionally, food‐derived polysaccharides can indirectly inhibit RAGE activation of subsequent signaling pathways by modulating the microbiome. For instance, eugenol polysaccharide, an α‐acidic heteropolysaccharide abundant in galactose and galactose glucuronide, reduces the abundance of inflammation‐associated bacteria such as Anabaena spp., Enteromonas spp., and Paraproteobacteria spp. While stimulating the growth of bacteria that produce short‐chain fatty acids (SCFAs). This microbial shift is accompanied by an increase in SCFA secretion. SCFAs can bind to the GPR43 receptor, inhibiting downstream histone deacetylase 3 and thereby downregulating the activation of the RAGE/NF‐κB and MAPK pathways. Moreover, SCFAs possess their own antioxidant and anti‐inflammatory properties, which contribute to the amelioration of colitis.^[^ ^156^ ^]^

### 5.3 Dietary Terpenoids

Terpenoids, a class of natural compounds formed by the head‐to‐tail linkage of isoprene units, are widely distributed across various organisms, including plants, animals, microorganisms, and marine life.^[^ ^157^ ^]^ As essential secondary metabolites, terpenoids play diverse roles in biological systems. Their structural diversity, ranging from simple monoterpenes to complex polyterpenes, and varied functional groups endow them with a wide array of biological activities and physicochemical properties. Due to these characteristics, terpenoids have significant application potential and growing market demand in fields such as medicine, food, and consumer products.^[^ ^158^ ^]^

Glycyrrhizin is a natural triterpene glycoconjugate extracted from the root of licorice (Glycyrrhiza spp.). It possesses a broad range of pharmacological properties, including antiviral, anti‐inflammatory, antitumor, and hepatoprotective activities, and is commonly used in the treatment of chronic hepatitis. Studies have shown that glycyrrhizin reduces the expression of HMGB1 in the cartilage of temporomandibular joint osteoarthritis, accompanied by a corresponding decrease in RAGE expression.^[^ ^159^ ^]^ Glycyrrhizin inhibits the phosphorylation of HMGB1, thereby suppressing its translocation and release.^[^ ^160^ ^]^ Furthermore, it has been reported to block the interaction between HMGB1 and RAGE by directly binding to HMGB1.^[^ ^161^ ^]^

Agastache rugosa (Fisch. et Meyer) O. Kuntze (A. rugosa, Labiatae) is a perennial aromatic herb from the Labiatae family, commonly used as a food spice and in traditional medicine for the treatment of colds, vomiting, and furuncles. β‐Caryophyllene (BCP, 4,11,11‐trimethyl‐8‐methylene‐bicyclo[7.2.0]undec‐4‐ene), a major active bicyclic sesquiterpene in A. rugosa, has been extensively studied for its biological activities, including anti‐inflammatory, antibacterial, and anticancer properties.^[^ ^162^ ^]^ Research has shown that BCP inhibits the release of serum HMGB1 in a GalN/LPS‐induced model of fulminant liver failure. Additionally, BCP mitigates GalN/LPS‐induced liver damage by suppressing TLR4 and RAGE‐mediated inflammatory signaling pathways.^[^ ^163^ ^]^

Recent studies have demonstrated that terpenoids, a major class of bioactive dietary components, can directly modulate RAGE signaling by competitively targeting its ligand‐binding domains. A notable example is geniposide, an iridoid glycoside terpenoid derived from Gardenia jasminoides, which acts as a high‐affinity RAGE antagonist. MST analysis revealed that geniposide binds to the V‐domain of RAGE with a Kd of 27.32 µM, exhibiting ≈17‐fold higher affinity than AGEs (Kd = 471.11 µM). Structural docking studies indicate that geniposide forms multiple hydrogen bonds with key residues within the V‐domain ligand‐binding pocket (N12, K15, W62, R66, E111, E153), thereby sterically obstructing the access of AGEs. This mechanism is further supported by co‐immunoprecipitation assays showing that geniposide disrupts the formation of the AGEs‐RAGE complex. As a result, geniposide effectively inhibits RAGE‐dependent signaling pathways, such as ERK and NF‐κB phosphorylation, and reduces the production of pro‐inflammatory cytokines, including TNF‐α and IL‐1β, both in vitro and in diabetic models. Unlike synthetic RAGE antagonists (e.g., FPS‐ZM1), which predominantly rely on hydrophobic interactions, geniposide exerts its effect through hydrogen‐bond‐driven binding, providing a structurally distinct and naturally derived scaffold for RAGE inhibition. These findings underscore the potential of dietary terpenoids to selectively disrupt RAGE‐ligand interactions and attenuate downstream pathological signaling.^[^ ^78^ ^]^

**Figure 7:**
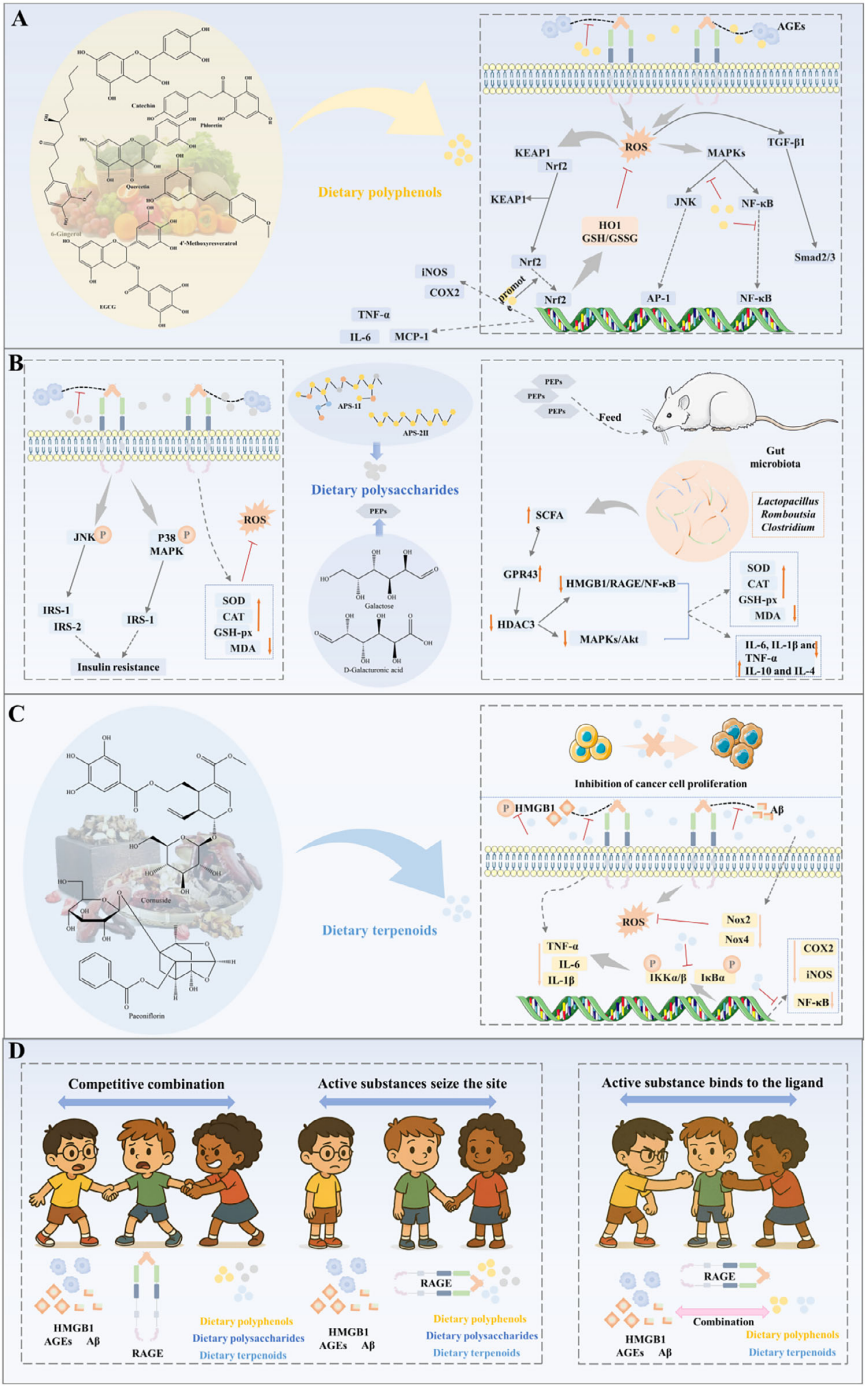
Inhibitory effects of polyphenols, polysaccharides and terpenoids upon RAGE binding with its ligands. A)Inhibitory effects of polyphenols upon RAGE binding with its ligands. B)Inhibitory effects of polysaccharides upon RAGE binding with its ligands. C)Inhibitory effects of terpenoids upon RAGE binding with its ligands. D)A schematic representation of the inhibition of RAGE‐ligand binding by dietary active ingredients.

Collectively, dietary polyphenols, polysaccharides, and terpenoids mitigate RAGE‐associated pathologies through complementary and synergistic mechanisms: 1) Suppression of ligand formation, primarily via carbonyl scavenging, which reduces the endogenous burden of AGEs and other reactive species; 2) Direct interference with RAGE‐ligand interactions, as exemplified by terpenoids such as geniposide, which binds the V‐domain of RAGE through hydrogen bonding with key residues (N12, K15, W62, R66, E111, E153), thereby sterically hindering AGEs docking; and 3) Inhibition of downstream pro‐inflammatory signaling, particularly via suppression of NF‐κB and ERK pathways—a mechanism shared across all three compound classes. Additionally, certain polysaccharides exert indirect regulatory effects by modulating gut microbiota composition, contributing to systemic downregulation of RAGE expression (**Figure** **7**). Despite these promising findings, high‐resolution structural insights into the precise binding interactions between natural compounds and RAGE remain limited. Future studies employing cryo‐EM and advanced binding epitope mapping are essential to elucidate these interactions at atomic detail and to accelerate the rational design of natural RAGE antagonists.

## 6 Green Processes and Anti‐Aging Products

### 6.1 Green Processing Techniques

**Figure 8:**
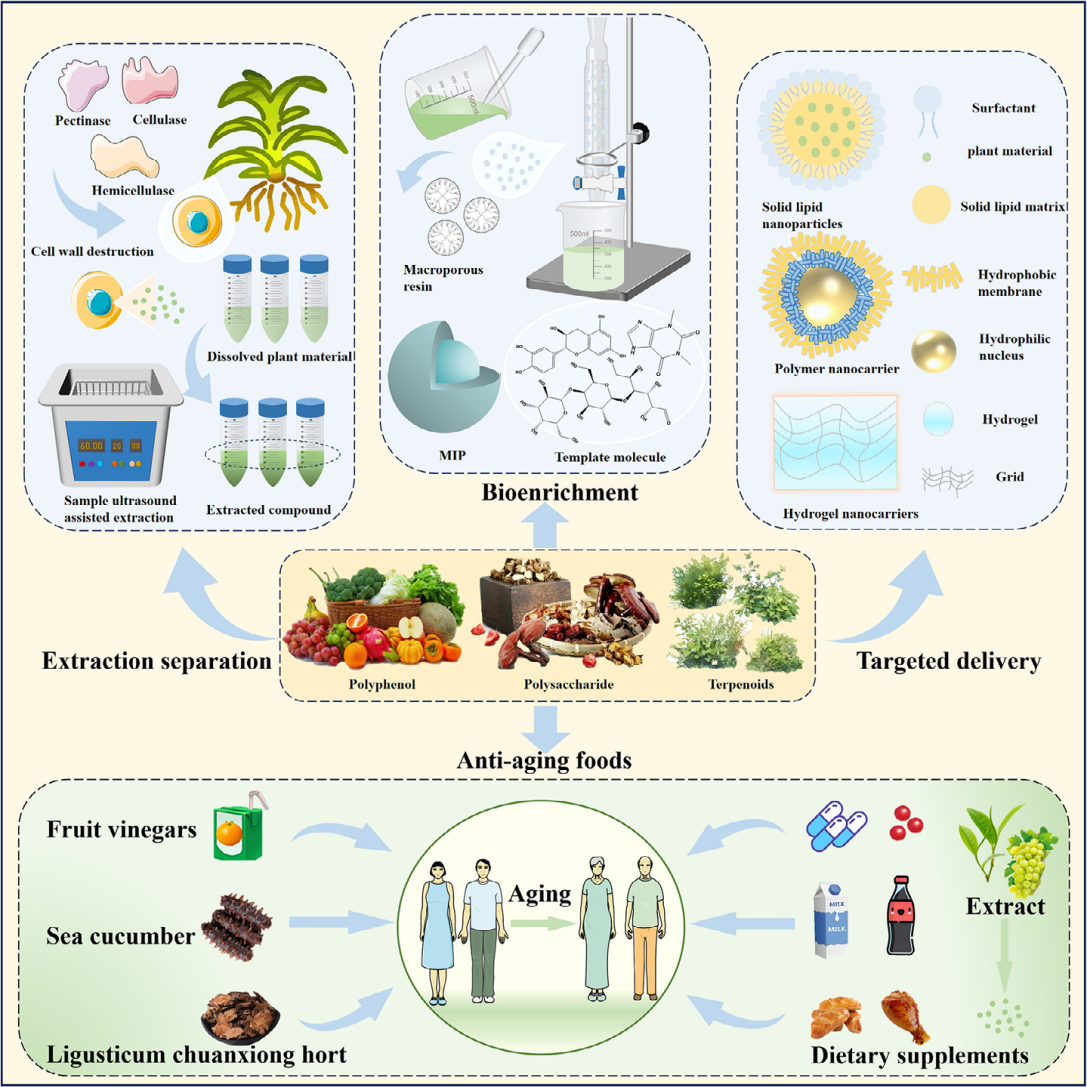
Green processes and anti‐aging foods.

Green processing techniques aim to address the challenges of the 21st century by creating environmentally sustainable, efficient, and low‐energy approaches for enhancing the bioactivity and delivery of plant‐derived compounds. Polyphenols, polysaccharides, and terpenoids, while exhibiting promising bioactivity against RAGE, often suffer from low stability and bioavailability due to the harsh conditions of the gastrointestinal tract, which limits their effectiveness in targeting RAGE directly. Additionally, the complex structures and low concentrations of these compounds in plants present further challenges in efficient extraction and delivery. This section will explore green extraction and enrichment techniques tailored to these compounds, as well as advanced targeted delivery systems designed to overcome these barriers. By optimizing stability and facilitating targeted delivery to RAGE, these green processing methods hold potential for applications across food, pharmaceuticals, and cosmetics, thereby supporting innovative strategies for mitigating RAGE‐mediated diseases (**Figure** **8**).

#### 6.1.1 Extraction and Separation

Conventional methods for extracting plant active ingredients such as polyphenols, polysaccharides, and terpenoids include hot water extraction, acid hydrolysis, chemical extraction, and maceration.^[^ ^164^, ^165^ ^]^ However, these techniques typically require large amounts of solvents, high temperatures, and extended extraction times. The harsh conditions associated with conventional extraction methods may also compromise the structural integrity of phytochemicals, thereby affecting their biological activity.^[^ ^166^, ^167^ ^]^ Enzymatic extraction is a widely adopted alternative that operates under mild conditions, minimizing the degradation of heat‐sensitive compounds.^[^ ^168^ ^]^ Studies have shown that enzymatic methods significantly enhance antioxidant activity compared to chemical extraction and are frequently employed to increase the yield of target substances derived from different sources.^[^ ^169^ ^]^ Furthermore, enzyme‐assisted extraction is regarded as a green process due to its environmentally friendly nature and reduced energy requirements. Common digestive enzymes, such as trypsin, papain and pectinase, which occur naturally in the human body, are often used to extract phytochemicals from fruits and plants.

Numerous natural products are sensitive to heat and may degrade during thermal extraction. Therefore, an ideal extraction method should maximize the yield of bioactive components while minimizing costs and processing time.^[^ ^170^ ^]^ Ultrasound‐assisted extraction (UAE) is an effective technique that enhances solvent penetration into plant cell walls, preventing potential chemical degradation of the target compounds.^[^ ^171^ ^]^ UAE operates on the principle of cavitation, whereby sound waves generate mechanical effects that create and collapse cavitation bubbles near or on the surface of the plant cell wall. This process enhances the contact surface area between the solid material and the solvent, facilitating the diffusion of cellular components and the solvent into the cells.^[^ ^172^ ^]^ The primary advantages of UAE include high extraction efficiency, the ability to operate at low temperatures, reduced processing time, and decreased solvent consumption.^[^ ^173^ ^]^

#### 6.1.2 Bio‐Enrichment

Isolating biologically active compounds from plant crude extracts is crucial for maximizing the therapeutic potential of natural resources. At present, various methods are employed to enrich bioactive substances, including centrifugal partition chromatography, preparative high‐performance liquid chromatography, high‐speed countercurrent chromatography, and membrane filtration. However, these methods often face significant drawbacks, such as complex operations, reliance on hazardous substances, high costs, and limitations for large‐scale production. In contrast, the macroporous resin‐based enrichment approach provides a practical, cost‐efficient, and eco‐friendly alternative.^[^ ^174^ ^]^ This method employs macroporous adsorbent resins (MARs), also known as fully porous resins or polymer adsorbents, which are characterized by their large pore mesh structure and absence of ion‐exchange groups. These porous cross‐linked polymers effectively enrich various substances. A variety of MARs with diverse physical properties, such as polarity, specific surface area, and pore size, are commercially available. These parameters can be flexibly selected based on the target compounds. With advantages such as excellent selective properties, high adsorption capacity, mild operating conditions, reusability, and scalability, macroporous resin methods have gained popularity for enriching natural products in recent years.^[^ ^175^ ^]^

Molecularly imprinted polymers (MIPs) are specialized polymers created through molecular imprinting technology (MIT).^[^ ^176^ ^]^ They feature specific recognition sites that are complementary to a chosen template in terms of size, shape, and binding motifs. This unique structure enables MIPs to selectively adsorb and enrich target bioactive components. For polyphenols, common templates like catechins can be used to enrich tea and grape polyphenols. In the case of polysaccharides, functional polysaccharides such as chitosan serve as templates, allowing for the selective isolation of target polysaccharides from complex mixtures by optimizing the pore structure of the polymers. Similarly, terpenoids can be efficiently enriched using MIPs.^[^ ^177^ ^]^ MIPs technology offers several advantages, including high selectivity, environmental friendliness, reusability, and thermal stability. These properties make MIPs widely applicable in various fields, including food, pharmaceuticals, and cosmetics. However, the technology still faces optimization challenges for large‐scale industrial applications.

#### 6.1.3 Targeted Delivery

Three types of plant active ingredients, polyphenols, polysaccharides, and terpenoids, exhibit beneficial effects on physiological functions. Nevertheless, the therapeutic potential of these bioactives remains underexplored because of challenges related to bioavailability, stability, and solubility, as well as issues with target specificity and insufficient residence time in the body. Recent advancements in nanocarrier technology for encapsulating therapeutic fractions have opened novel approaches for improving the therapeutic potential of bioactives.^[^ ^178^, ^179^ ^]^ By embedding bioactive components within nanoparticles, we can effectively prevent their degradation while enhancing their bioactivity.^[^ ^180^ ^]^ Nano‐embedding not only improves solubility and stability but also provides site specificity, prolonged circulation times, and desirable release profiles. Various nanocarrier systems have been developed for this purpose, including solid lipid nanoparticles (SLNPs), polymer nanoparticles, and hydrogels. These innovations represent significant progress toward maximizing the therapeutic applications of bioactive compounds.^[^ ^181^ ^]^

SLNPs are colloidal systems formed by dispersing solid lipid matrices, including fatty acids, specific glycerides, and steroids, dispersed with stabilizing surfactants. The resulting particle sizes typically range from 50 to 10 000 nm.^[^ ^182^ ^]^ In this system, the active compounds are encapsulated within a central core of solid lipid. SLNPs are versatile nanocarriers suitable for various routes of administration, providing significant stability to the loaded compounds against degradation.^[^ ^183^ ^]^ Polymer‐Based Nanocarriers, also referred to as submicron solid particles, consist of colloidal vesicles constructed from biodegradable polymers. These can be either natural (such as proteins or polysaccharides) or synthetic.^[^ ^184^ ^]^ The final sizes of these nanocarriers range from 10 to 1000 nm. Their structure allows for effective encapsulation of bioactive substances, enhancing their therapeutic efficacy and bioavailability.^[^ ^185^ ^]^ Polymer‐based nanocarriers can take various forms depending on fabrication techniques and encapsulation modes, including nanospheres, nanocapsules, dendritic polymers, and micelles. Nanospheres are characterized by a uniform dispersion of loaded drugs within a polymer matrix, where the active compounds are integrated throughout the structure.^[^ ^186^ ^]^ In contrast, nanocapsules encapsulate the drug within small cavities formed by polymer membranes, allowing for a more localized release of the therapeutic agent. Polymeric micelles are created by the assembly of amphiphilic copolymers, which can be either diblock or triblock structures. The drug is encapsulated within the micelle's hydrophobic core. This design facilitates improved stability in blood circulation and allows for better control over drug release, extending the circulation time of the delivery system in the body. Hydrogel nanocarriers consist of hyperbranched colloidal polymers capable of absorbing large quantities of liquid and swelling in an aqueous environment. In this system, the drug is entrapped within the reticular cross‐links of the gel, remaining inactive until a sufficient stimulus, such as temperature, pH, ionic strength, or light, triggers its release.^[^ ^187^ ^]^ The key advantages of using hydrogels include a significant increase in drug bioavailability, controlled release mechanisms, and the versatility of administration routes.

### 6.2 Anti‐Aging Foods

Studies have shown that dietary polyphenols, polysaccharides, and terpenoids possess anti‐aging properties by scavenging free radicals and inhibiting the formation of AGEs during glycation reactions. Furthermore, these bioactive compounds may mitigate aging‐related processes by preventing the interaction between RAGE and its ligands. As research in this field continues to expand, polyphenols, polysaccharides, and terpenoids have found widespread application in the development of anti‐aging foods. In addition to enhancing the health benefits and functional value of these products, these compounds may also contribute to alleviating aging‐related pathological conditions associated with RAGE, such as neurodegenerative diseases like AD and Parkinson's disease. Consequently, the research and development of dietary polysaccharides, polyphenols, and terpenoids hold significant potential for practical applications and offer valuable scientific insights.

For instance, fruit vinegars, which are abundant in polyphenols, serve as excellent dietary sources of AGEs inhibitors. A recent study investigated eight varieties of fruit vinegars have been analyzed for their inhibitory effects on fluorescent AGEs, with orange vinegar demonstrating the highest inhibition rate. The findings have revealed that orange vinegar, along with its primary components, catechin, epicatechin, and p‐coumaric acid, significantly reduced levels of ROS, RAGE, NADPH, and inflammatory markers in Caco‐2 cells. This research provides a theoretical basis for the application of orange vinegar as an AGEs inhibitor.^[^ ^188^ ^]^

Sea cucumber (WS) is a nutrient‐rich food containing a wide range of natural bioactive compounds, including 55 biologically active substances that promote human health. Among these, crude polysaccharides, proanthocyanidins, and anthocyanins have been shown to reduce oxidative stress, thereby delaying aging and preventing age‐related diseases. Dietary supplementation with WS has been reported to alleviate aging‐related symptoms, such as enhancing cognitive functions like learning and memory, reducing neuropathological changes, and improving immune function. Additionally, research indicates that aged mice exhibit significant alterations in gut microbiota and metabolic profiles compared to normal mice, and WS may help restore these parameters to healthier levels. By modulating gut microbiota and metabolic pathways, WS demonstrates promising anti‐aging effects, positioning it as a potential functional food for aging prevention.^[^ ^189^ ^]^

Ligusticum chuanxiong Hort (CX) is a widely cultivated medicinal and edible plant native to Sichuan Province, China. In addition to its medicinal uses, CX is commonly consumed as a food ingredient; its tender leaves are used in salads or stir‐fried dishes, while its rhizome is incorporated into teas, soups, and alcoholic beverages. Studies have identified various phytochemicals in CX, including terpenoids, organic acids, terpenes, and polyphenols. However, further research is needed to determine which specific compounds are most responsible for its anti‐aging and neuroprotective effects. Existing studies have demonstrated that extracts from both the leaves and rhizomes of CX exhibit significant antioxidant, anti‐aging, and neuroprotective properties.^[^ ^190^ ^]^

With the ongoing advancements in anti‐aging research, scientists are broadening their exploration of natural compounds with potential health benefits. In addition to the direct consumption of naturally bioactive‐rich foods such as WS and CX, researchers are isolating key active components, including polyphenols, polysaccharides, and terpenoids, from various sources. These bioactive compounds are being formulated into dietary supplements using advanced technologies such as nanoparticle delivery systems,^[^ ^191^ ^]^ hydrogels, and probiotic encapsulation.^[^ ^192^ ^]^ The integration of these functional ingredients into food products shows significant promise for the development of innovative anti‐aging dietary interventions aimed at promoting longevity.

## 7 Challenges and Future Perspectives

### 7.1 The Challenges of Research on Dietary Active Components Targeting RAGE

Studies on the inhibition of RAGE by dietary active ingredients face several challenges. The complex matrices of food and the interactions among various components can lead to substantial alterations in the bioavailability, metabolic pathways, and mechanisms of action of these active ingredients in vivo. Consequently, this increases the complexity and difficulty of such studies. RAGE functions as a multiligand receptor with substantial variations in affinity and binding kinetics toward different ligands. However, a comprehensive structure‐function explanation detailing how dietary active ingredients modulate these receptor‐ligand interactions remains lacking. Despite the preliminary evidence from in vitro and animal studies, the absence of systematic and rigorous human clinical trials has hindered the extrapolation and practical application of these findings. Therefore, future studies are urgently needed to address these challenges and to incorporate multi‐omics approaches and emerging biological tools, aiming to provide a more comprehensive understanding of the potential and mechanisms by which dietary active ingredients modulate RAGE signaling.

#### 7.1.1 The Complex Composition of Food Hinders the Research of RAGE

Given the frequent occurrence of synergistic interactions among various components within complex food systems, which may either enhance or diminish the functional properties of individual components, it is crucial to employ multi‐omics techniques to elucidate the in vivo pathways and regulatory mechanisms of these active ingredients. Numerous studies have demonstrated that specific dietary active ingredients can interfere with the binding of RAGE to its various ligands, thereby inhibiting RAGE‐mediated inflammation and oxidative stress. This suggests significant potential for these ingredients in the field of anti‐aging. Therefore, it is crucial to investigate the conformational relationships of dietary active ingredients. Each ingredient's unique structural characteristics, including molecular weight, molecular conformation, chain length, branching degree, substituent type, and substitution position, can significantly influence its binding affinity to target proteins as well as its anti‐aging efficacy.^[^ ^193^ ^]^ Even minor structural differences can result in significant fluctuations in biological activity. Therefore, conducting in‐depth studies on structural modifications and conformational relationships is of great value in the screening and optimization of anti‐aging functional factors. The biological effects of individual food ingredients are often influenced by the synergistic actions of other components. Such synergistic effects may arise through mechanisms including enhanced absorption, stabilization of activity, or simultaneous action on the same target. The study of the structural characteristics and kinetic behaviors of these active ingredients in composite food matrices, including their stability, release profiles, bioavailability, and in vivo metabolic transformations, provides an important theoretical foundation and technical support for developing anti‐aging products.

#### 7.1.2 The Structure‐Activity Relationship Among Receptor‐Ligands and Active Ingredients

RAGE plays a significant role in the aging process. However, whether the accumulation of RAGE ligands and the binding affinity of RAGE to its ligands vary across the kidney, liver, and skeletal muscle during aging remains unclear. Studies have shown that the age‐related accumulation pattern of RAGE ligands is organ‐dependent. The binding strength of RAGE to its ligands increases with age in the kidney and liver but remains unchanged in skeletal muscle.^[^ ^194^ ^]^ However, systematic studies and clear molecular‐level explanations of the detailed mechanisms of RAGE and its ligands, as well as how they synergistically contribute to the progression of aging, are still lacking. An in‐depth analysis of the specific interaction patterns between RAGE and its ligands in various organs, along with their associated regulatory networks, holds great scientific significance for elucidating aging‐related signaling pathways and identifying potential intervention targets. Dietary active ingredients have been shown to inhibit the binding of RAGE to its various ligands, potentially slowing the aging process by suppressing RAGE‐mediated pathways such as chronic inflammation and oxidative stress. However, the specific molecular mechanisms underlying the tripartite interactions among dietary active ingredients, RAGE, and its ligands remain unclear. These include the precise binding sites on the receptor, binding affinities, and the dynamic processes influencing downstream signaling. Therefore, there is an urgent need to systematically analyze the 3D conformations, binding kinetics, and downstream effects of these complexes using multidimensional approaches, including structural biology, molecular modeling, and multi‐omics analyses. In addition, systematic investigations are required to assess the formation and residual levels of potentially toxic by‐products generated during extraction, processing, or storage. Particular attention should be paid to the potential toxicity associated with high‐dose or long‐term intake, in order to ensure the safety and feasibility of these compounds for use in functional food development and clinical applications.

The therapeutic application of dietary bioactive compounds demands thorough evaluation of their toxicity profiles, particularly at pharmacological or supra‐dietary doses. Polyphenols such as EGCG and quercetin exhibit dose‐dependent adverse effects. Notably, they significantly impair iron absorption via metal chelation, reducing absorption by over 60% at doses of 100 mg, and can cause clinically relevant drug interactions through inhibition of cytochrome P450 enzymes and OATP transporters. For instance, co‐administration of green tea has been shown to reduce the oral bioavailability of nadolol by ≈85%. In addition, endocrine‐disrupting effects have been reported; high intake of isoflavones, equivalent to consuming three quarts of soy milk per day, has been linked to gynecomastia and breast tissue hyperplasia in men.^[^ ^195^ ^]^ Polysaccharides derived from microbial and algal sources introduce additional challenges. Sulfated variants, such as those from Ulva species, are metabolized by gut microbiota into hydrogen sulfide, which impairs mitochondrial function in colonic epithelial cells.^[^ ^196^ ^]^ Similarly, spirulina‐derived polysaccharides exhibit delayed cytotoxicity, with significant effects observed at >800 µg mL^−1^ in RAW264.7 cells after 48 h of exposure.^[^ ^197^, ^198^ ^]^ Terpenoids present notable toxicity concerns due to narrow therapeutic windows. Triptolide, for example, exhibits a four‐fold margin between efficacious (0.45 g kg^−1^) and hepatotoxic doses (1.8 g kg^−1^). Its organ‐specific toxicity is attributed to structural features such as the C‐14 hydroxyl group, which contributes to metabolic suppression, such as 50% CYP3A4 downregulation and reproductive toxicity, including an 80.65% reduction in sperm motility at 0.2% dietary concentrations.^[^ ^199^ ^]^

These findings underscore the need for a comprehensive safety assessment framework encompassing: 1) long‐term toxicity monitoring, including hepatic and renal function, even for compounds with high acute tolerance; 2) evaluation of compound‐microbiota‐host metabolic interactions, particularly relevant for sulfated polysaccharides and glycoside‐rich polyphenol supplements; and 3) recognition of population‐specific vulnerabilities. These include iron‐deficient individuals, who are susceptible to polyphenol‐induced malabsorption; patients with inflammatory bowel disease, who may be sensitive to fermentable polysaccharide byproducts; and individuals of reproductive age, who may face endocrine or reproductive risks from terpenoid exposure. Future research should prioritize the development of threshold‐based dosing guidelines anchored in mechanistic toxicity markers, such as oxidative stress induction, enzymatic inhibition, and microbial dysbiosis, to ensure the safe and translational application of dietary bioactives in anti‐aging strategies.

#### 7.1.3 Lack of Support from Population or Human Experiments

In recent years, increasing attention has been directed toward understanding the molecular mechanisms of aging and exploring potential intervention strategies. While certain clinical drugs have demonstrated anti‐aging activity, concerns remain regarding their safety, long‐term tolerability, and potential side effects, limiting their widespread application. Against this backdrop, dietary active ingredients have attracted extensive attention as safer and more sustainable anti‐aging strategies. Various dietary compounds have demonstrated notable anti‐aging effects in multiple animal models, including Caenorhabditis elegans, fruit flies, and mice. Their anti‐aging properties are attributed to multiple mechanisms, including enhanced antioxidant capacity, regulation of age‐related gene expression, and improvement of immune function.^[^ ^200^ ^]^ However, it is important to acknowledge that animal models have inherent limitations in aging research and cannot fully replicate the complex physiological and metabolic environment of humans. The specific types and doses used in animal models may not accurately represent human intake, and whether these effects translate similarly in the human body remains to be fully confirmed. Human dietary sources are typically more complex and diverse, involving interactions among multiple food components and dietary habits. These factors are often challenging to replicate accurately in animal studies.^[^ ^201^ ^]^ The anti‐aging effects of dietary active ingredients are often influenced by various non‐dietary factors, including genetic background, lifestyle, microbiome composition, and environmental exposures. Together, these complex factors determine their mechanisms of action and intervention outcomes in the human body. Currently, the absence of systematic and long‐term human randomized controlled trials limits the clinical extrapolation of existing research findings. Therefore, it is imperative to comprehensively evaluate the anti‐aging potential and safety of dietary active ingredients in real‐world populations to facilitate their practical application.

While preclinical studies provide compelling evidence for the role of dietary bioactive compounds such as polyphenols, polysaccharides, and terpenoids in modulating RAGE signaling and attenuating aging‐related pathologies, translation into clinical practice necessitates rigorous human validation. Notably, current clinical data specifically addressing RAGE‐targeted modulation by these dietary constituents in the context of aging remain limited and are still in the early stages of development. Nevertheless, an expanding body of clinical trials has explored the broader health benefits of these compounds and diets rich in them, often assessing endpoints closely associated with RAGE activation and age‐related disease mechanisms, including systemic inflammation, oxidative stress, metabolic dysregulation, and vascular dysfunction. These findings suggest promising translational potential but highlight the urgent need for targeted, mechanistic clinical studies to directly evaluate the efficacy of natural compounds as modulators of the RAGE signaling axis in human aging.

For instance, a randomized crossover trial in overweight and obese individuals demonstrated that the polyphenol combination trans‐resveratrol and hesperetin (tRES‐HESP) significantly suppressed RAGE expression in peripheral blood mononuclear cells by 37% (p < 0.05), while concurrently improving insulin sensitivity and inflammatory biomarkers.^[^ ^202^ ^]^ This study provides the first clinical evidence supporting the modulation of the RAGE pathway by dietary polyphenols. In parallel, a phase I pharmacokinetic trial investigating the polysaccharide‐based compound low‐anticoagulant heparin (ODSH) revealed that it achieves therapeutic anti‐inflammatory plasma concentrations (30‐60 µg mL^−1^) without significant anticoagulant activity. Notably, ODSH effectively blocked RAGE‐ligand interactions with HMGB1 and S100 proteins, underscoring the translational potential of polysaccharide‐based RAGE antagonists.^[^ ^203^ ^]^ In contrast, no clinical trials to date have directly evaluated dietary terpenoids for RAGE‐targeted interventions. Collectively, these studies begin to bridge the gap between preclinical findings and human application, offering proof‐of‐concept evidence for the efficacy of dietary RAGE modulators. Nonetheless, larger and longer‐term clinical trials across diverse populations remain essential to validate their sustained efficacy and safety in aging‐related disease prevention.

Despite promising findings, significant gaps remain in the current body of evidence. Many existing clinical trials are of relatively short duration, utilize supraphysiological doses delivered via supplements rather than whole food sources, and often focus on isolated compounds rather than complex dietary patterns. Moreover, direct assessments of RAGE pathway activity, such as measurements of sRAGE, esRAGE, circulating ligands, or tissue‐specific RAGE signaling, are frequently lacking. Heterogeneity in study designs, participant demographics (e.g., age, health status, genetic background), and intervention protocols further complicates cross‐study comparisons and limits the generalizability of findings. Critically, there remains a scarcity of long‐term, large‐scale clinical trials specifically designed to investigate the modulation of RAGE‐mediated aging processes as primary outcomes. Addressing these limitations is essential to establish robust translational frameworks for dietary RAGE‐targeted interventions in human aging.

### 7.2 Future Prospects of Anti‐Aging Research Targeting RAGE

Given the current challenges, future research should aim to comprehensively explore the structure‐activity relationships, biological pathways, and clinical applicability of dietary bioactive compounds in modulating RAGE activity. The ultimate goal is to integrate these compounds into daily diets, develop precise dietary strategies, and establish long‐term, safe, and effective aging interventions.

#### 7.2.1 Elucidating the Mechanisms of Active Compounds in Complex Food Matrices

To address the limitations caused by complex food matrices, future research should focus on analyzing the in vivo metabolic pathways, biological fate, and absorption of active compounds using advanced techniques such as multi‐omics integration, metabolite tracing, and in situ detection.^[^ ^204^ ^]^ These approaches can provide a more accurate picture of the bioavailability, distribution, and transformation of bioactive compounds in the body. In particular, the interactions between multiple food components and their synergistic or antagonistic effects should be investigated to uncover key regulators in real dietary conditions. Additionally, developing efficient delivery systems, such as encapsulation, nanocarriers, or hydrogels, can improve the stability and targeted release of active compounds, helping to overcome the limitations of instability and low bioavailability. These technological advances will support the effective application of RAGE‐targeting functional foods.

#### 7.2.2 Clarifying the Structure‐Activity Relationship and Molecular Interactions

To better understand how dietary compounds inhibit RAGE‐ligand interactions, future work should combine structural biology, computational simulation, and molecular docking techniques to elucidate the specific binding sites and conformational changes of RAGE and its ligands.^[^ ^205^ ^]^ Analyzing how compound structural features. Such as functional groups, stereochemistry, chain length, and branching, which affect their binding affinity to RAGE will help identify molecular markers that predict anti‐RAGE activity. Furthermore, mapping ligand‐specific signaling responses in different tissues can reveal the tissue‐specificity of dietary compounds. This knowledge will contribute to the design of targeted interventions for RAGE‐related aging processes. The application of high‐throughput screening and synthetic modification may further optimize natural compounds and enhance their anti‐aging activity.

#### 7.2.3 Advancing Human Clinical Research and Precision Nutrition Strategies

To unequivocally validate the RAGE‐targeting potential observed preclinically and establish effective dietary strategies for healthy aging, large‐scale, well‐controlled, long‐term human clinical trials are essential. These studies should evaluate the safety, dosage, and efficacy of dietary bioactive compounds in realistic dietary patterns and across different population groups. Longitudinal studies and population cohorts will help identify the long‐term benefits and possible risks of sustained intake. At the same time, combining data on genetics, gut microbiota, metabolomics, and lifestyle factors will promote the development of precision nutrition strategies. Personalized dietary recommendations tailored to individual physiological and genetic profiles will maximize the benefits of RAGE‐targeted functional compounds.^[^ ^206^ ^]^ In the long run, establishing dietary patterns and public health guidelines based on RAGE modulation mechanisms can provide a scientific foundation for preventing chronic diseases and promoting healthy aging.

## 8 Summary and Prospects

RAGE plays a crucial role in the pathogenesis of several aging‐related diseases, making it essential to study the interaction between RAGE and its ligands both in vivo and in vitro in order to inhibit the activation of downstream signaling pathways. This paper provides a general overview of the processes and considerations involved in preparing RAGE using the E. coli protein expression system. It summarizes the binding kinetics of RAGE and its ligands in vitro, utilizing techniques such as SPR, ITC, MST, and NMR. The paper also briefly discusses the signaling mechanisms activated by RAGE upon ligand binding and highlights the roles of dietary polyphenols, polysaccharides, and terpenoids in RAGE activation. It reveals that many biologically active components of dietary inhibit the generation and accumulation of RAGE ligands in vivo, thereby blocking the activation of downstream cellular signaling pathways. However, current research predominantly concentrates on ligand interactions, regulation of RAGE expression, and downstream signaling pathways, while studies exploring the dynamic conformational changes of RAGE's transmembrane domains, the topological characteristics of its ligand‐binding pocket, and the structural mechanisms underlying receptor dimerization remain limited. Future research can leverage cutting‐edge techniques such as cryo‐EM to reconstruct high‐resolution structures of RAGE‐ligand complexes, and crosslinking mass spectrometry to capture how pH fluctuations or redox changes within the cellular microenvironment influence the extracellular conformation of RAGE. Additionally, integrated analytical strategies combining hydrogen‐deuterium exchange mass spectrometry and MD simulations can be employed to systematically elucidate the binding modes of functional dietary components such as polyphenols and polysaccharides with key amino acid residues of RAGE. Critically, translating these structural insights into practical interventions necessitates prioritization of two key areas: 1) establishing optimal dosing regimens for bioactive dietary compounds, such as polyphenol–polysaccharide combinations, that achieve sustained RAGE inhibition in vivo; and 2) investigating synergistic interactions among natural compounds, including terpenoid–polyphenol blends, to enhance therapeutic efficacy while minimizing required dosages and associated toxicity risks. This structural and topological understanding will lay a solid molecular foundation for the development of functional food ingredients that specifically target and modulate the structural domains of RAGE, advancing precision nutrition approaches for aging‐related disease prevention. Finally, the paper discusses green processing techniques and potential product applications for these three types of active ingredients. In the future, RAGE could serve as a key target for exploring natural bioactive compounds in plant‐based diets that regulate or intervene in aging‐related diseases by inhibiting RAGE. This could help identify additional natural anti‐aging ingredients and provide a theoretical foundation for the development of functional foods and natural RAGE inhibitors.

## Conflict of Interest

The authors declare no conflict of interest.
